# Putative LysM Effectors Contribute to Fungal Lifestyle

**DOI:** 10.3390/ijms22063147

**Published:** 2021-03-19

**Authors:** Marta Suarez-Fernandez, Ana Aragon-Perez, Luis Vicente Lopez-Llorca, Federico Lopez-Moya

**Affiliations:** 1 Laboratory of Plant Pathology, Department of Marine Sciences and Applied Biology, University of Alicante, 03690 Alicante, Spain; anaaragonperez1@gmail.com (A.A.-P.); lv.lopez@ua.es (L.V.L.-L.); 2 Laboratory of Plant Pathology, Multidisciplinary Institute for Environmental Studies (MIES) Ramon Margalef, University of Alicante, 03690 Alicante, Spain

**Keywords:** biocontrol agents, endophytism, fungal effectors, fungal lifestyles, LysM motifs, pathogenicity, phylogeny, *Pochonia chlamydosporia*

## Abstract

Fungal LysM effector proteins can dampen plant host–defence responses, protecting hyphae from plant chitinases, but little is known on these effectors from nonpathogenic fungal endophytes. We found four putative LysM effectors in the genome of the endophytic nematophagous fungus *Pochonia chlamydosporia* (Pc123). All four genes encoding putative LysM effectors are expressed constitutively by the fungus. Additionally, the gene encoding Lys1—the smallest one—is the most expressed in banana roots colonised by the fungus. Pc123 Lys1, 2 and 4 display high homology with those of other strains of the fungus and phylogenetically close entomopathogenic fungi. However, Pc123 Lys3 displays low homology with other fungi, but some similarities are found in saprophytes. This suggests evolutionary divergence in Pc123 LysM effectors. Additionally, molecular docking shows that the NAcGl binding sites of Pc123 Lys 2, 3 and 4 are adjacent to an alpha helix. Putative LysM effectors from fungal endophytes, such as Pc123, differ from those of plant pathogenic fungi. LysM motifs from endophytic fungi show clear conservation of cysteines in Positions 13, 51 and 63, unlike those of plant pathogens. LysM effectors could therefore be associated with the lifestyle of a fungus and give us a clue of how organisms could behave in different environments.

## 1. Introduction

*Pochonia chlamydosporia* (Goddard) [1] (Hypocreales, Clavicipitaceae) is a nematophagous fungus that parasites eggs and females of root-knot and cyst nematodes [1,2,3]. *P. chlamydosporia* is known to display a tritrophic lifestyle because it is also an endophyte and a weak saprophyte [4,5,6]. *P. chlamydosporia* is a relevant endophytic biocontrol agent (EBCA) because it promotes growth and induces defences in plants [7,8,9,10,11,12]. This fungus is both an EBCA and a biofertiliser capable of promoting flowering and yield [13]. For these activities, the fungus should colonise plant roots. In this process, *P. chlamydosporia* has to face plant defences. The plant immune system detects pathogen-associated molecular patterns (PAMPs) and microbe-associated molecular patterns (MAMPs). Pattern-triggered immunity (PTI) is then induced [14,15], and pathogenesis-related (PR) proteins are secreted. These plant defence mechanisms can also be induced by chitin-derived oligosaccharides such as chitosan [16]. On the other hand, fungal endophytes and plant pathogens secrete lysine motif (LysM) effector proteins that bind to chitin in their cell walls, masking fungi to avoid degradation by plant chitinases [17]. LysM effectors are essential for fungal hyphae protection, as they bind to chitin-derived oligosaccharides released from the cell wall of the fungus [18,19,20]. This union blocks the generation of chitin oligosaccharides (common MAMPs and PAMPs), which are very strong PTI inducers [21], and therefore, PR proteins are not induced. Thus, fungal LysM effectors block plant defences and plant hormone signalling [18,20,22,23,24,25], allowing fungi to get into the cells of the plant root.

LysM effectors are small secreted proteins containing LysM motifs exclusively [26]. They bind N-acetylglucosamine (GlcNAc) polymers [27,28]. These proteins show a high percentage of cysteines, which stabilise the molecular structure through disulphide bridges. LysM motifs have approximately 50 amino acids (aa) and a characteristic βααβ spatial structure in which two β-strands draw an antiparallel β-sheet [20,29,30,31]. LysM motifs have been found in bacteria [29], fungi [20], plants [32] and animals [23].

The large presence of LysM effectors in fungi has resulted in a variety of LysM motifs [33]. They can be classified based on their cysteine residue patterns into bacterial/fungal and fungal-specific ones [23,24,26]. The bacterial/fungal group presents one or no cysteine within the LysM motif. The fungal-specific group possesses three cysteines within the LysM motif and an extra cysteine very close to its origin. All these features, as well as the fact that more than 95% of those described are of bacterial origin [26], prove that the study of these proteins is vital to understand mutualism and parasitism.

In this work, we characterise and model the main putative LysM effectors from the endophytic nematophagous fungus Pc123 and prove that genes encoding for those proteins are expressed. We also compare putative LysM effectors of 57 fungi with diverse lifestyles, including endophytes and plant pathogens. The evolutionary study of these proteins can give clues to the behaviour that an organism may have, as well as explain why some fungi behave as endophytes or pathogens.

## 2. Results

### 2.1. Pc123 Has Four Putative LysM Effectors

**Figure 1:**
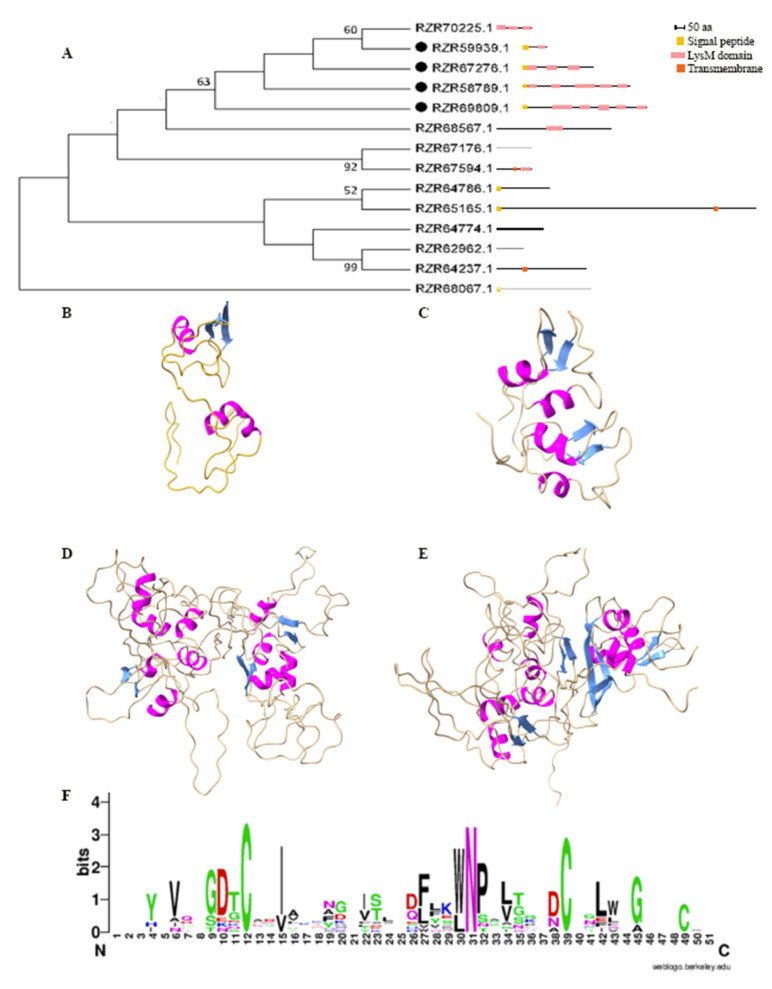
Putative LysM effectors of the nematophagous fungus Pc123. (**A**) Phylogeny of the 14 Pc123 proteins containing LysM domains. Dots indicate putative LysM effectors. (**B**–**E**) Molecular modelling Pc123 putative LysM effectors: (**B**) Pc123Lys1; (**C**) Pc123Lys2; (**D**) Pc123Lys3; (**E**) Pc123Lys4. (**F**) WebLogo analysis of domains of Pc123 putative LysM effectors. Phylogenetic analysis was performed in MEGA X v10.1 by aligning the sequences using ClustalW v2.0.12, with a Maximum Likelihood, 1500 Bootstraps and the Jones–Taylor–Thornton (JTT) method. All models were performed with Modeller.

Fourteen proteins with LysM motifs are found in the *Pochonia chlamydosporia* 123 (Pc123) genome (NCBI BioProject PRJNA68669; Table S1). Four of them meet all requirements to be considered putative LysM effectors. They possess signal peptides, a high percentage of cysteines (over 3%) and only contain LysM domains. Proteins with high evolutionary similarity to any of the putative LysM effectors have been discarded for not meeting some of the established requirements (e.g., RZR70225.1). The phylogeny of putative Pc123 LysM domain-containing proteins shows that Pc123 putative LysM effectors cluster together (Figure 1A and Table 1). Protein modelling shows that the four putative LysM effectors predicted in the Pc123 genome show a characteristic βααβ spatial structure. Two β-strands draw an antiparallel β-sheet. This may be essential for its biological activity (Figure 1B–E). Pc123 Lys1 and Pc123 Lys2 are homologous to LysM domain-containing proteins from entomopathogenic fungi, mostly *Metarhizium* spp. and *Beauveria* spp. (Tables S2 and S3). Fungi from the genera *Metarhizium* and *Beauveria* also use the same mechanisms to colonise plants and act as endophytic BCAs like *P. chlamydosporia*. These entomopathogenic fungi genera are close phylogenomically to Pc123 [34]. Unlike them, Pc123 Lys3 displays low homology with LysM-containing proteins of other organisms (<50%), most of them saprophytes (Table S4). This correlates to the fact that *P. chlamydosporia* is also found as a saprophyte in the soil [35]. Most similarities are found with *Fusaria* and *Aspergilli*. Finally, Pc123 Lys4 has 100% homology with a sequence from *P. chlamydosporia* strain 170, but other similar sequences belong mostly to *Colletotrichum* spp. (Table S5), a phytopathogenic fungus. These four putative Pc123 LysM effectors could give us a clue about the evolution of its tritrophic lifestyle.

Modeller (v9.24) provides structures with all predicted domains for Pc123 Lys1 and Lys2. According to the Pfam, Superfamily and Gene3D databases, the Lys1 protein sequence only has a LysM domain approximately at the C-terminal end. Furthermore, according to these databases, the protein includes signal peptides from Positions 1 to 20. In the protein sequence of Lys2, the Pfam and Superfamily databases identify two LysM domains, while the Gene3D library recognised the presence of three of these domains. All of them are placed approximately in the central region of the ORF coding sequence. All databases also determine the existence of signal peptides from Positions 1 to 22. For modelling Pc123 Lys3, only five LysM motifs of the six predicted by Pfam, Superfamily and Gene3D could be detected. Three of them are clearly identified by their beta strands and alpha helices. In addition, two pairs of alpha helices are observed, which would indicate the position of the two other motifs. For Pc123 Lys4, five motifs are detected by Modeller, as well as with Pfam, Superfamily and Gene3D. Four of the motifs can be well recognised by their alpha and beta composition.

WebLogo v2.8.2 analysis (Figure 1F) shows that in all LysM domains of Pc123 putative LysM effectors, cysteines (Positions 12, 39 and 49) and the Trp-Asn-Pro/Leu-Asn-Pro (WNP/LNP) set (Positions 30–32) are conserved. This may indicate that most domains of these putative effectors belong to the fungal group [26].

### 2.2. Putative LysM Effectors May Be Associated with the Lifestyle of a Fungus

**Figure 2:**
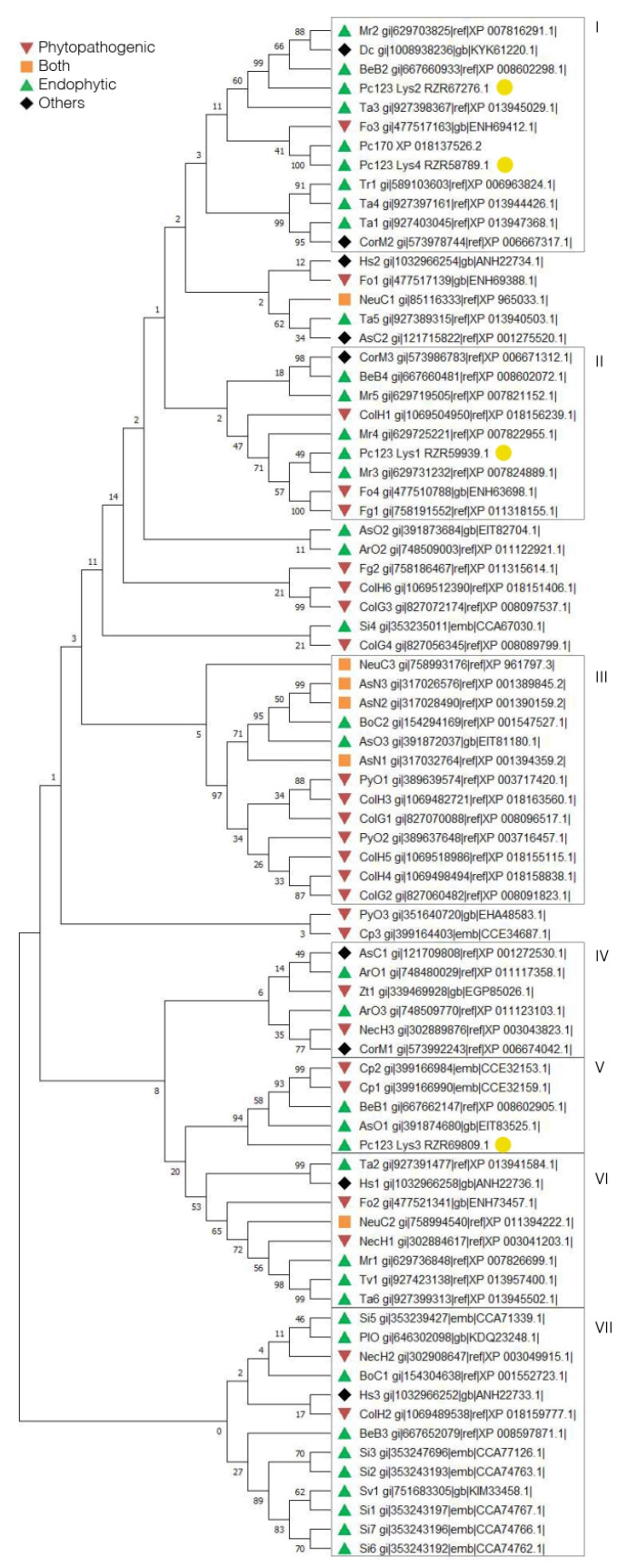
Fungal endophytes and phytopathogens can be grouped according to their putative LysM effector sequences. This phylogeny of putative LysM effectors contains 27 different organisms and 81 sequences. Phylogeny is grouped into lifestyles: endophytes, pathogens and both. Phylogenetic analysis was performed in MEGA X by aligning the sequences using ClustalW, with a Maximum Likelihood tree, 1500 Bootstraps and the JTT method. Abbreviations are listed in Table 1. Pc123 putative LysM effectors are marked with yellow dots.

Fifty-seven organisms with diverse lifestyles were selected to search for their putative LysM effectors (Table S1). Within these organisms, 27 contained proteins that met the requirements to be putative LysM effectors (Table 1). Phylogenetic analyses of sequences were performed to understand the evolution of these proteins. Putative LysM effectors (Figure 2) from phytopathogenic (Cluster III) and those of endophytic fungi (Clusters I and VII) mostly lay in separate clusters. Clusters II, IV and VI include putative LysM effectors from both types of fungi. Putative LysM effectors from Pc123 lay in Clusters I, II and V. This heterogenicity may suggest a divergent evolution in these proteins. The phylogenetic analysis shows that Pc123 Lys1 (Cluster II) is similar to *Metarhizium robertsii* LysM effectors. This agrees with the BLASTp results described above (Tables S2–S5). For Pc123 Lys2 (Cluster I), similarities are found with putative LysM effectors from *M. robertsii*, *Beauveria bassiana* and *Trichoderma arthroviride*. The major homology presented by Pc123 Lys3 (cluster V) is with putative LysM effectors of *B. bassiana* and *Aspergillus oryzae*. Finally, Pc123 Lys4 (Cluster I) displays a 99% homology with Pc170-1. Fungi such as *Beauveria*, *Aspergillus*, *Arthrobotrys* and *Trichoderma* can also be found in this cluster. A phylogenetic study, based on the analysis of the Pc123 LysM domain sequence identified in the four Pc123 putative LysM effectors described above, shows that all domains cluster together with that described in endophytic fungi (Figure S1). These results support the hypothesis that these proteins play a key role in the ability of this fungus to colonise plants.

### 2.3. Patterns of LysM Motifs May Reflect Fungal Lifestyle

**Figure 3:**
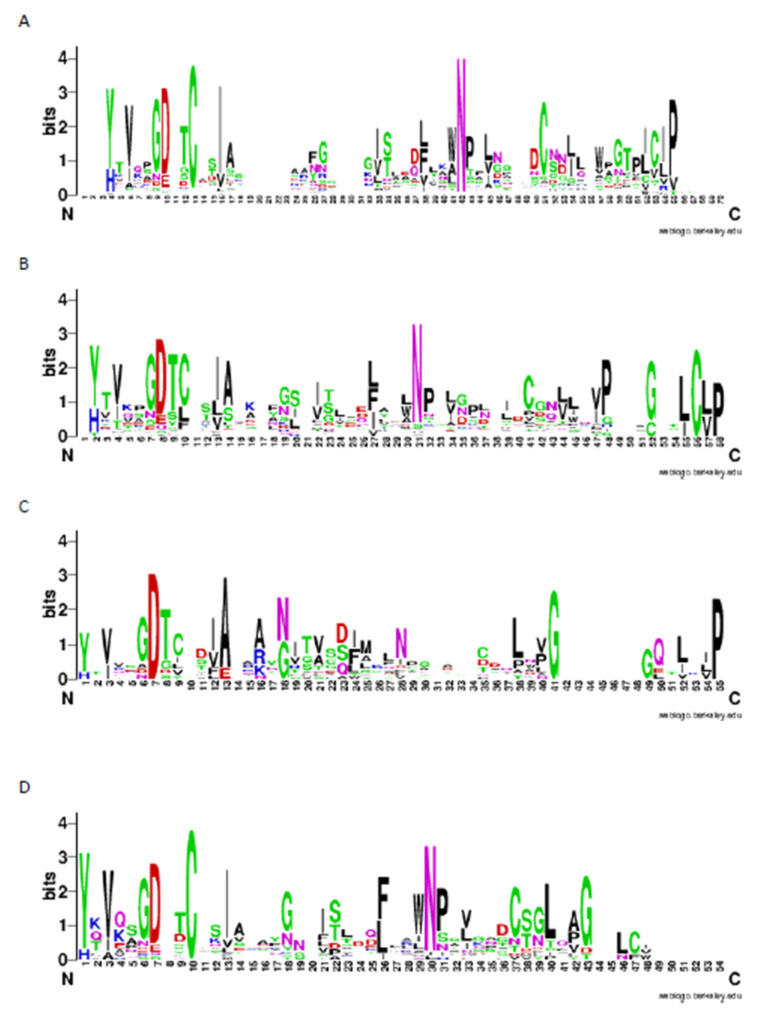
Amino acid conservation of LysM domains. Classification of LysM domains according to their lifestyle using WebLogo. (**A**) Endophytic lifestyle; (**B**) phytopathogenic lifestyle; (**C**) both lifestyles; (**D**) others. Letters correspond to the amino acid code that is used with the International Nucleotide Sequence Database, one letter code.

According to Cys classification, 29 LysM domain sequences have no cysteines, 72 LysM domain sequences have only one cysteine, 75 LysM domain sequences have two cysteines and 49 LysM domain sequences have three cysteines. One LysM domain sequence from *Drechmeria coniospora* has four cysteines, and one LysM domain sequence from *Fusarium oxysporum* has five cysteines. Organisms were divided into four groups according to the bibliography, even if these are not their major lifestyles: endophytes, phytopathogens, both and others. For endophytes, WebLogo analyses clearly show the conservation of cysteines in Positions 13, 51 and 63, as well as the conservation of chitin-binding-related amino acid groups (Gly-Asp-Cys-Thr or GDCT structure, Positions 9–13). Asn (N) is also conserved at Position 42. The Trp-Asn-Pro (WNP) structure of the same position, found in many LysM domains, is clearly noticeable (Figure 3A). On the other hand, phytopathogenic fungi show conservation in some cysteines, but it seems to be only remarkable Positions 10, which belongs to the GDCT motif of chitin binding, and 56. The N of Position 31, as in endophytes, is preserved (Figure 3B). Fungi that have both endophytic and phytopathogenic lifestyles only have preserved the GDCT domain. In this case, these domains have few cysteines, and the most preserved amino acids are Ala (A) in Position 13, Gly (G) in 41 and Pro (P) in 55. In Position 28, N is slightly conserved, belonging in the two previous cases to WNP or Leu-Asn-Pro (LNP) domains, although in this case, it is not as conserved as in the two cases mentioned above (Figure 3C). Finally, the group of fungi that could not be classified as endophytes and/or pathogens, of which *Aspergillus clavatus*, *Cordyceps militaris*, *Drechmeria coniospora* and *Hirsutella sinensis* are part, has a different conservation of amino acids from the previous ones, with the domain of binding to chitin Gly-Asp-Cys (GD-C) from Positions 6 to 10. Two Cysteines in Position 10 and 37, as well as the already mentioned WNP motif (Positions 29–31). Other amino acids that did not stand out in the previous groups are also very much preserved: Ile (I) in Position 13, Leu (L) in Position 40 and Gly (G) in Position 43 (Figure 3D).

### 2.4. Molecular Docking

**Figure 4:**
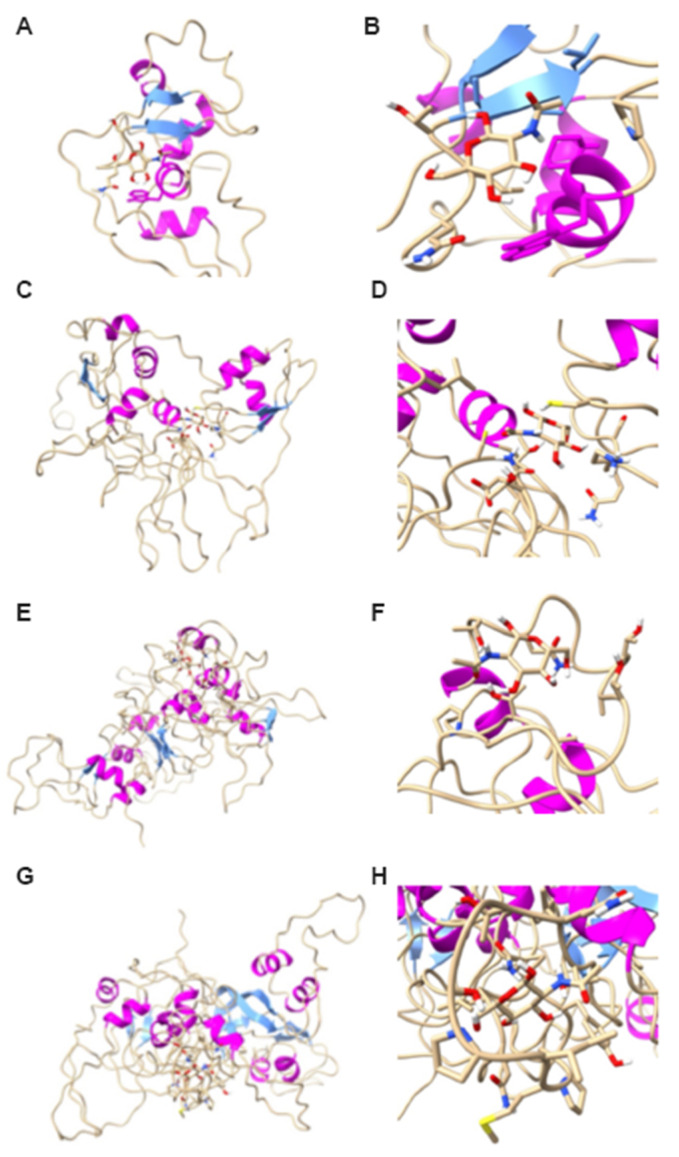
Molecular docking of putative LysM effectors of Pc123. GlcNAc binding model to all putative Pc123 LysM effectors. (**A**) Pc123Lys1; (**B**) broadening of the GlcNAc binding site to Pc123Lys1; (**C**) Pc123Lys2; (**D**) broadening of the GlcNAc binding site to Pc123Lys2; (**E**) Pc123Lys3; (**F**) broadening of the GlcNAc binding site to Pc123Lys3; (**G**) Pc123Lys4; (**H**) broadening of the GlcNAc binding site to Pc123Lys4.

We have analysed the association of NAcGl with the putative LysM effectors of Pc123 using molecular docking (Figure 4). Predicted NAcGl binding sites of putative Pc123 Lys 1, 2, 3 and 4 effectors have 10, 17, 10 and 14 residues, respectively. Putative Pc123 LysM effectors share NAcGl putative binding sites from diverse organisms (Table 2).

The union site of Pc123 Lys1 possesses 10 residues. It shares three types of residues with both the chitinase A from *Pteris ryukyunensis* and the effector ECP6 from *Clavidosporium fulvum* sites of union to GlcNAc and chitin, respectively (Thr, Ala and Gly). Additionally, it shares another four with only the site of union of the effector ECP6; these are: Leu, Pro, Val and Lys. The union site of the putative effector Pc123 Lys2 possesses 17 different residues, 4 of them are shared with both the chitinase A and the ECP6 effector (Gly, Asn, Thr and Ser). Another four are shared just with the ECP6 effector (Lys, Leu, Phe and Asp). Moreover, one last residue is shared only with the chitinase A (Cys). The union site of the putative effector Pc123 Lys3 has 10 residues, three are shared with both the chitinase A and the effector ECP6 (Thr, Ala and Gly) and three are shared only with the effector ECP6 (Val, Asn and Pro). Finally, the union site of the putative effector Pc123 Lys4 possesses 14 residues, from which 4 are shared with both proteins (Thr, Ile, Ser and Gly), 2 with the effector ECP6 (Leu and Pro) and one with the chitinase A (Tyr). All similarity percentages of the 4 putative effectors of Pc123 are shown in Table S6.

Additionally, in Pc123 Lys2, the areas between the α helix and β strands of two LysM motifs, in which sites of union to chitin in the effector ECP6 have been found, are clearly involved in the union with GlcNAc. In the putative effector Pc123 Lys3 also, areas next to two adjacent α helices from a LysM motif (just identified by its α helix) are involved in the union to the substrate. The same seems to happen in Pc123 Lys4, with two LysM motifs detected by their α helix. ProSa and Rampage results can be consulted in Figure S2.

### 2.5. Genes Encoding Putative LysM Effectors Are Expressed

**Figure 5:**
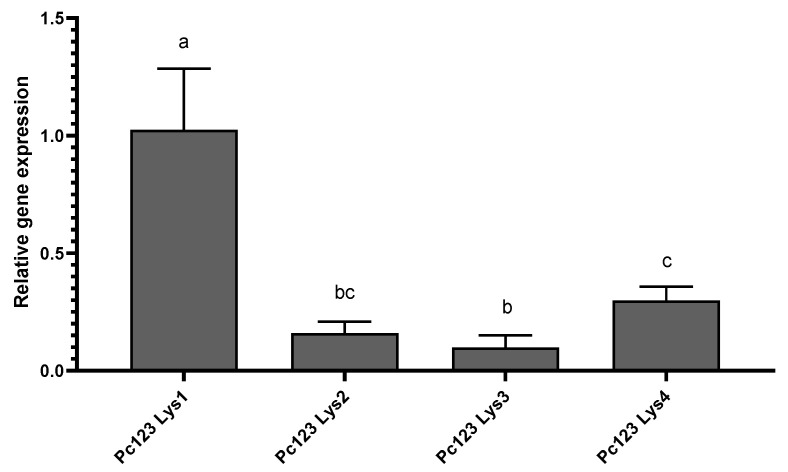
Putative Pc123 LysM effectors expression in banana roots. All four genes encoding putative LysM effectors are expressed constitutively by the fungus. Pc123 Lys1 is the most expressed gene. Genes encoding Pc123 Lys2, 3 and 4 are expressed ca. 6.25-, 10- and 3.35-fold less, respectively. One-way ANOVA analysis was performed (*p* < 0.05). Letters (a,b,c,d) indicate significant differences.

All four genes encoding putative LysM effectors are expressed constitutively by Pc123 (Figure 5). Pc123 Lys1 is the most expressed gene. With respect to it, Lys2 is expressed ca. 6.25-fold less, Lys3 ca. 10-fold less and Lys4 ca. 3.35-fold less. No significant differences in expression were found for genes encoding LysM effectors when Pc123 colonises banana roots at four days (Figure S3).

## 3. Discussion

*P. chlamydosporia* is an endophytic BCA of plant parasitic nematodes [36]. In this work, we found that *P. chlamydosporia* (strain 123) genome [34] encodes four putative LysM effectors. LysM effectors are small peptides containing LysM domains for binding NAcGl in chitin and peptidoglycan [37]. These domains can be found in other chitin-binding proteins such as chitinases [38]. Putative effectors Pc123 Lys1 and Lys4 have homologous sequences with the isolate Pc170 recently sequenced [39]. Although Pc170 was isolated from eggs from the root-knot nematode *Meloidogyne incognita* in China [39] and Pc123 was isolated from *Heterodera avenae* eggs in South-West Spain [40], both genomes show high synteny. More than 80% of the Pc123 genome matches the Pc170 genome with 96.45% identity [39]. However, the NCBI database shows that the Pc170 genome only encodes two putative LysM effectors. This difference may suggest the differential evolution of chitin shielding in strains of *P. chlamydosporia*. Molecular docking analyses reveal that Pc123 putative LysM effectors share Positions for chitin binding with a fern [31] and filamentous fungal pathogen [20]. This may suggest that the basic structures for the target binding of LysM effectors are evolutionary conserved. Endophytic nematophagous fungi such as Pc123 may colonise plant roots using these effectors to avoid plant defences. In this respect, the life cycle of endoparasites of nematodes is mostly related to their hosts [41]. Furthermore, *H. rosiliensis* and other nematode endoparasites such as *D. coniospora* [42] display low or no endophytic behaviour in barley roots [43]. These fungi are usually obligated nematode parasites. Their genomes encode very low or no putative LysM effectors (Table 1).

Nematophagous fungi with alternative lifestyles [44], including endophytism such as *A. oligospora* (nematode-trapping fungus) *P. chlamydosporia* (nematode egg and female parasite) and *Pleurotus ostreatus* (toxin-producing nematophagous fungus) [10,45,46], encode a larger number of putative LysM effectors than endoparasites. Entomopathogenic fungi such as *Beauveria bassiana* and *Metarhizium robertsii* are also endophytes [47,48] and encode a high number of putative LysM effectors. *Trichoderma* spp., which are both mycoparasites and endophytes [49,50,51], also encode a large number of putative LysM effectors. These fungi presumably interact with GlcNAc from their hosts. Pc123 interacts with chitin in the nematode eggshell. Similarly, entomopathogenic organisms must deal with GlcNAc residues from insect cuticles. *Trichoderma* spp. deal with chitin residues from the wall of their target fungi. Additionally, all these organisms detect, which chitin they produce. This could explain the large number of putative LysM effectors they encode. Moreover, this could be the reason why all putative Pc123 LysM effectors are expressed constitutively. Pc123 Lys1—the smallest one—is the most expressed. It could attach to the fungal wall, hiding its own chitin. Basal expression of putative LysM effectors of Pc123 has also been detected when the fungus infects nematode eggs [52], which is related to the basal expression of putative LysM effectors observed in this work. According to our study, *Serendipita indica* (*Piriformospora indica*) is the fungal endophyte [53] sequenced to date with the most putative LysM effectors encoded. Fungi with LysM effectors use them to colonise plants, but they might use them to take part in other processes, such as parasitism or pathogenicity. Saprophytic fungi also encode a large number of putative LysM effectors. These proteins may protect hyphae of saprophytes from chitinases released in root exudates [54], which are present in the rhizosphere. Moreover, *Aspergillus oryzae* [55] and *A. niger* [56] can behave as endophytes and, as well as saprophytes. *A. niger* and *Neurospora crassa*, well-known saprophytes, can also behave as plant pathogens under certain conditions [57,58]. This flow between lifestyles shows how easily fungi can modify their behaviour according to the environment.

In our study, putative Pc123 LysM effectors lay in different phylogenetic clusters (I, II and V), which may suggest an evolutionary divergence. The theory of balanced antagonism [59] states that fungal endophytism or plant pathogenicity depends on the host–pathogen balance, e.g., a pathogen in a host may be an endophyte in another plant. LysM effectors take part in a strategy to avoid plant immunity [60]. However, the plant is still able to respond to the presence of the fungus. In previous works, it has been shown that plant root colonisation by Pc123 induces the expression of plant genes related to hormone biosynthesis [11]. In fact, LysM effectors from endophytes and pathogens should be similar since they perform a parallel role. However, in this work, we have found that LysM motifs have undergone divergent evolution in endophytes vs. plant pathogenic fungi. If plant immunity is a key factor to mutualism and parasitism (or pathogenicity), this may explain why chitin shielding by LysM effectors seems to be an evolutionary trend. Unlike plant pathogens, LysM motifs from endophytes have three conserved cysteines. Some fungi, like *A. niger*, have low or no Cys in most of its LysM domains, which would mean their saprophytic lifestyle is determined by LysM domains similar to the bacterial group [26], while fungi with s higher Cys content in LysM domains, such as endophytes, have LysM domains similar to those of the fungal bacterial group. The fact that facultative endophytes have coexisted and coevolved alongside chitin, and pathogens have not had this close contact, might be the key to the differences between LysM effectors of both lifestyles.

In conclusion, we show that LysM effectors may reflect the lifestyle of a fungus, which makes them an important tool in endophytism and pathogenicity studies. In future studies, it will be possible to locate possible LysM effectors in situ and inquire into the effects they may have on the plant immune system. Furthermore, this work serves as a basis for future research on the sustainable use of BCAs for protecting crops in two ways: the use of plant defences inducing microorganisms in agriculture and the use of endophytic microorganisms as fertilisers and enhancers of plant development (plant hormone producers).

## 4. Materials and Methods

### 4.1. Identification of Putative LysM Effectors

Putative LysM effectors were detected in the nematophagous fungus Pc123 (PRJNA68669) and in 57 other genomes sequenced fungi (Table S1), including endophytes and plant pathogens. The presence of a signal peptide, LysM domains, peptide length and a high cysteine percentage were scored. LysM domains, length and signal peptide were determined using HMMERscan. Cysteine percentage was calculated manually. The result after the screening is shown in Table 1. The comparative analysis of putative LysM effectors from other *P. chlamydosporia* strains and other fungi was performed using BLASTp (https://blast.ncbi.nlm.nih.gov/).

### 4.2. Identification of Protein Domains

To identify all protein domains present in putative LysM effector protein sequences, HMMER web server tool hmmscan (v3.2.1) was used [61]. For this purpose, different databases were employed: Pfam v33.1 [62], CATH-Gene3D v4.3 [63], TIGRFAMs v15.0 [64], Superfamily v1.75 [65] and PIRSF [66] and TreeFam v9 [67].

### 4.3. Three-Dimensional Structures

Modeller v9.24 [68] was used to model the three-dimensional structure of the protein sequences from the genes identified as putative Pc123 LysM effectors. All structures were generated with the protein models of the fungal effector Ecp6 from *Cladosporum fulvum* [20] and two chitinase A, both from *Pterys rykyunesis* [69] and *Equisetum arvense* [70], as templates. Additionally, for the structural modelling of Pc123 Lys3 and Pc123 Lys4, the structure of the rice chitin receptor OsCEBiP from *Oryza sativa* [71] was also used (PDB database access numbers: 4b8v, 4pxv, 5bum and 5jcd; https://www.rcsb.org/; Identity Percentages are shown in Table S6). Five models were generated per each putative Pc123 LysM effector. The structural integrity of the model with the lowest DOPE was analysed by ProSa [72] and the “Rampage” Ramachandran plot utility [73]. The best Pc123 Lys1 model yielded a ProSa Z-score of −2.72 and showed 5 residues as outliers in the Ramachandran plot. The best Pc123 Lys2 model yielded a ProSa Z-score of −0.56 and 11 residues as outliers in the Ramachandran plot. For Pc123 Lys3, a ProSa Z-score of 0 and 23 residues as outliers in the Ramachandran plot were obtained. Finally, the best model for Pc123 Lys4 yielded a ProSa Z-score of −1.68, showing 31 residues as outliers in the Ramachandran plot. All the models were visualised with UCSF-Chimera v1.14 [74].

### 4.4. Phylogenetic Analyses and Molecular Docking

All phylogenetic trees were performed using the MEGA X v10.1 software [75]. Sequence alignments were performed using ClustalW. Phylogenetic trees were constructed using the Maximum Likelihood method and the Jones–Taylor–Thornton (JTT) model [76]. Statistical support for each of the branches was determined by 1500 permutations (Bootstrap) [77]. The results of the LysM motif alignment were also used to perform a Logo sequence analysis through the WebLogo program [78]. Molecular docking was performed using AutoDock VINA v1.1.2 [79] by applying default parameters.

### 4.5. qRT-PCR

Pc conidia (final concentration: 10^6^ conidia·mL^−1^) were inoculated into 100 mL flasks each containing 20 mL of Czapeck Dox broth medium [80]. Flasks were incubated at 25 °C with shaking at 120 rpm. After five days, mycelia were recovered by filtration through Miracloth (Calbiochem) and washed twice with sterile distilled water (SDW). Pc mycelia (ca. 0.2 g) were inoculated axenically into 100 mL flasks, each containing 20 mL of Minimal Medium (MM) [81] or in Magenta Boxes^TM^ (Sigma, Munich, Germany), each containing 50 mL of MM and a banana plantlet. Plants were maintained at 24 °C, with 60% relative humidity and a 16:8 h light/darkness photoperiod, with 100 rpm shaking, for 4 days. To extract RNA, three plant roots were collected for each extraction. Three replicates were obtained per treatment. RNA was extracted using the RNeasy Plant Mini Kit (Qiagen, Hilden, Germany). qRT-PCR was performed using the FastStart Universal SYBR Green Master (Roche, Basel, Switzerland) mix in a final volume of 15 μL, using 0.25 µM of each primer. Reactions were performed in triplicate in a Step One Plus (Applied Biosystems, Foster City, CA, US.) following these steps: 95 °C 10 min, followed by 40 cycles of 95 °C for 15 s and 60 °C for 45 s. Primers used for qRT-PCRs are shown in Table S7. *P. chlamydosporia* allantoate permease [82], glyceraldehyde-3-phosphate dehydrogenase [83] and ß-tubulin [80] were used as housekeeping genes.

## Figures and Tables

**Table 1:** Consensus table of 27 organisms containing putative LysM effectors in their proteome. NCBI Reference Sequence, length, percentage of cysteines (% Cys), number of LysM domains (predicted with HMMERscan) and the sequence name to refer to each effector are included.

Organism	Sequence Name	Length	%Cys	LysM Domains	Name Putative Effector
*Pochonia chlamydosporia* 123	gi|1576959602|gb|RZR59939.1|	123	4.88	1	Pc123_Lys-1
	gi|1576967440|gb|RZR67276.1|	377	5.57	3	Pc123_Lys-2
	gi|1576970205|gb|RZR69809.1|	665	4.96	6	Pc123_Lys-3
	gi|1576958349|gb|RZR58789.1|	577	3.81	5	Pc123_Lys-4
*Pochonia chlamydosporia* 170	gi|1240655211|ref|XP_018137526.2|	601	4.16	5	Pc170-1
	gi|1069526848|ref|XP_018144528.1|	123	4.88	1	Pc170-2
*Arthrobotrys oligospora* ATCC 24927	gi|748480029|ref|XP_011117358.1|	677	5.02	6	ArO-1
	gi|748509003|ref|XP_011122921.1|	387	4.13	4	ArO-2
	gi|748509770|ref|XP_011123103.1|	701	4.71	5	ArO-3
*Aspergillus clavatus* NRRL 1	gi|121709808|ref|XP_001272530.1|	527	4.93	5	AsC-1
	gi|121715822|ref|XP_001275520.1|	343	4.66	4	AsC-2
*Aspergillus niger* CBS 513.88	gi|317032764|ref|XP_001394359.2|	235	2.55	4	AsN-1
	gi|317028490|ref|XP_001390159.2|	223	3.59	3	AsN-2
	gi|317026576|ref|XP_001389845.2|	228	3.51	3	AsN-3
*Aspergillus oryzae* 3.042	gi|391874680|gb|EIT83525.1|	488	3.89	2	AsO-1
	gi|391873684|gb|EIT82704.1|	400	3.50	2	AsO-2
	gi|391872037|gb|EIT81180.1|	228	3.51	3	AsO-3
*Beauveria bassiana* ARSEF 2860	gi|667662147|ref|XP_008602905.1|	563	4.80	4	BeB-1
	gi|667660933|ref|XP_008602298.1|	384	5.73	3	BeB-2
	gi|667652079|ref|XP_008597871.1|	258	2.33	2	BeB-3
	gi|667660481|ref|XP_008602072.1|	167	7.19	2	BeB-4
*Botryotinia fuckeliana* = *Botrytis cinerea* B05.10	gi|154304638|ref|XP_001552723.1|	239	1.67	1	BoC-1
	gi|154294169|ref|XP_001547527.1|	227	3.52	4	BoC-2
*Colletotrichum graminicola* M1.001	gi|827070088|ref|XP_008096517.1|	153	4.58	2	ColG-1
	gi|827060482|ref|XP_008091823.1|	154	3.90	2	ColG-2
	gi|827072174|ref|XP_008097537.1|	96	4.17	1	ColG-3
	gi|827056345|ref|XP_008089799.1|	262	4.58	2	ColG-4
*Colletotrichum higginsianum* IMI 349063	gi|1069504950|ref|XP_018156239.1|	686	4.52	4	ColH-1
	gi|1069489538|ref|XP_018159777.1|	335	5.37	2	ColH-2
	gi|1069482721|ref|XP_018163560.1|	170	3.53	2	ColH-3
	gi|1069498494|ref|XP_018158838.1|	176	3.98	2	ColH-4
	gi|1069518986|ref|XP_018155115.1|	164	1.22	1	ColH-5
	gi|1069512390|ref|XP_018151406.1|	93	4.30	1	ColH-6
*Cordyceps militaris* CM01	gi|573992243|ref|XP_006674042.1|	455	4.84	3	CorM-1
	gi|573978744|ref|XP_006667317.1|	541	4.25	6	CorM-2
	gi|573986783|ref|XP_006671312.1|	187	6.95	2	CorM-3
*Drechmeria coniospora*	gi|1008938236|gb|KYK61220.1|	407	5.90	4	Dc-1
*Fusarium graminearum* PH-1	gi|758191552|ref|XP_011318155.1|	221	4.52	3	Fg-1
	gi|758186467|ref|XP_011315614.1|	178	6.18	2	Fg-2
*Fusarium oxysporum* (FOCTR1)	gi|477517139|gb|ENH69388.1|	298	4.03	3	Fo-1
	gi|477521341|gb|ENH73457.1|	423	5.67	3	Fo-2
	gi|477517163|gb|ENH69412.1|	265	4.53	3	Fo-3
	gi|477510788|gb|ENH63698.1|	218	4.59	2	Fo-4
*Metarhizium anisopliae* = *Metarhizium robertsii* ARSEF 23	gi|629736848|ref|XP_007826699.1|	588	4.42	2	Mr-1
	gi|629703825|ref|XP_007816291.1|	403	5.46	4	Mr-2
	gi|629731232|ref|XP_007824889.1|	125	4.80	2	Mr-3
	gi|629725221|ref|XP_007822955.1|	127	4.72	1	Mr-4
	gi|629719505|ref|XP_007821152.1|	175	5.14	2	Mr-5
*Fusarium solani* = *Nectria hematococca* mpVI77-13-4	gi|302884617|ref|XP_003041203.1|	434	5.07	3	NecH-1
	gi|302908647|ref|XP_003049915.1|	354	5.37	2	NecH-2
	gi|302889876|ref|XP_003043823.1|	453	4.86	2	NecH-3
*Neurospora crassa* OR74A	gi|85116333|ref|XP_965033.1|	265	4.53	3	NeuC-1
	gi|758994540|ref|XP_011394222.1|	460	4.78	2	NeuC-2
	gi|758993176|ref|XP_961797.3|	540	2.22	3	NeuC-3
*Magnaporthe oryzae* = *Pyricularia oryzae* 70–15	gi|389639574|ref|XP_003717420.1|	162	3.70	2	PyO-1
	gi|389637648|ref|XP_003716457.1|	285	2.11	2	PyO-2
	gi|351640720|gb|EHA48583.1|	276	4.71	1	PyO-3
*Piriformospora indica* = *Serendipita indica*	gi|353243197|emb|CCA74767.1|	527	3.80	5	Si-1
	gi|353243193|emb|CCA74763.1|	418	5.74	4	Si-2
	gi|353247696|emb|CCA77126.1|	163	4.29	2	Si-3
	gi|353235011|emb|CCA67030.1|	171	4.68	1	Si-4
	gi|353239427|emb|CCA71339.1|	174	5.17	2	Si-5
	gi|353243192|emb|CCA74762.1|	654	4.59	8	Si-6
	gi|353243196|emb|CCA74766.1|	361	4.43	4	Si-7
*Pleurotus ostreatus*	gi|646302098|gb|KDQ23248.1|	133	6.77	2	PlO-1
*Trichoderma atroviride* IMI 206040	gi|927403045|ref|XP_013947368.1|	746	4.29	5	Ta-1
	gi|927391477|ref|XP_013941584.1|	544	4.78	2	Ta-2
	gi|927398367|ref|XP_013945029.1|	345	4.64	3	Ta-3
	gi|927397161|ref|XP_013944426.1|	511	4.11	3	Ta-4
	gi|927389315|ref|XP_013940503.1|	241	5.39	3	Ta-5
	gi|927399313|ref|XP_013945502.1|	443	4.97	2	Ta-6
*Trichoderma reesei* QM6a	gi|589103603|ref|XP_006963824.1|	473	4.44	4	Tr-1
*Trichoderma virens* Gv29-8	gi|927423138|ref|XP_013957400.1|	440	5.00	2	Tv-1
*Serendipita vermifera MAFF 305830*	gi|751683305|gb|KIM33458.1|	141	4.96	2	Sv-1
*Zymoseptoria tritici IPO323*	gi|339469928|gb|EGP85026.1|	97	4.12	1	Zt-1
*Hypocrella siamensis*	gi|1032966258|gb|ANH22736.1|	312	4.81	2	Hs-1
	gi|1032966254|gb|ANH22734.1|	323	3.72	3	Hs-2
	gi|1032966252|gb|ANH22733.1|	86	4.65	1	Hs-3
*Claviceps purpurea 20.1*	gi|399166990|emb|CCE32159.1|	689	5.22	6	Cp-1
	gi|399166984|emb|CCE32153.1|	688	5.23	5	Cp-2
	gi|399164403|emb|CCE34687.1|	90	2.22	1	Cp-3

**Table 2:** Molecular docking. Amino acids that bind to the NAcGl substrate with the greatest probability for each of the putative LysM effectors of Pc123. Common amino acids that mediate substrate binding in Ecp6 and Pc123 are underlined and those common to the chitinase A from *Pteris ryukyunensis* and Pc123 are marked in bold.

Name	Bind.Energy [kcal/mol]	Dissoc. Constant [pM]	Contacting Receptor Residues
Pc123 Lys1	>−5.9760	>41,642,224.00	>GLN 13 LEU 14 **THR 15 ALA 16** VAL 17 VAL 18 LYS 98 TRP 99 PRO 101 **GLY 102**
>Pc123 Lys2	>−6.3100	>23,697,836.00	>CYS 83 **GLY 84 ASN 85** THR 111 **THR 112 SER 113** GLN 114 LYS 115 LEU 157 GLN 218 **CYS 269** THR 270 GLY 271 PHE 288 ASP 289 THR 290 GLN 309
>Pc123 Lys3	>−5.6040	>78,021,360.00	>VAL 428 THR 429 THR 590 ASN 591 **THR 596 ALA 597** THR 598 GLY 601 **GLY 602** PRO 604
Pc123 Lys4	−6.4030	20,255,304.00	LEU 283 GLN 284 **TYR 361** GLN 362 **THR 492 ILE 493** GLN 494 THR 495 **SER 497** PRO 498 ILE 499 MET 500 PRO 501 **GLY 502**

## Data Availability

Not applicable.

## Supplementary Materials

The following are available online at https://www.mdpi.com/1422-0067/22/6/3147/s1. Figure S1. LysM domains of putative effectors of different organisms are grouped according to their way of life. Phylogeny of putative LysM effectors belonging to 27 different organisms, only LysM domains (232 in total). Phylogeny is grouped into lifestyles: endophytes, phytopathogens, both and others. Phylogenetic analysis was performed in MEGA X by aligning the sequences using ClustalW, with a Maximum Likelihood, 1500 Bootstraps, JTT method. Abbreviations are listed in Table 1. Figure S2. Quality of protein models. A, Pc123 Lys1 ProSa; B, Pc123 Lys1 “Rampage” Ramachandran data; C, Pc123 Lys2 ProSa; D, Pc123 Lys2 “Rampage” Ramachandran data; E, Pc123 Lys3 ProSa; B, Pc123 Lys3 “Rampage” Ramachandran data; F, Pc123 Lys4 ProSa; G, Pc123 Lys4 “Rampage” Ramachandran data. Figure S3. Putative Pc123 LysM effectors are expressed in banana. *vcp1* (used as positive control) is overexpressed during root colonisation. There were not found significant differences in putative LysM expression between banana roots colonised by Pc123 and Pc123 growing in minimal medium. Table S1. NCBI BioProject and number of proteins containing LysM domains of *P. chlamydosporia* 123 and 56 other organisms with diverse lifestyles. Table S2. BLASTp of the putative effector Pc123 LysM 1. There are similarities with sequences of entomopathogenic fungi. Table S3. BLASTp of the putative effector Pc123 LysM 2. There are similarities with sequences of entomopathogenic fungi. Table S4. BLASTp of the putative effector Pc123 LysM 3. There are hardly any significant similarities with sequences of other fungi, but saprophytic fungi are among them. Table S5. BLASTp of the putative effector Pc123 LysM 4. There are similarities with sequences of phytopathogenic fungi. Table S6. Percentage of model identity of the 4 putative LysM effectors of Pc123 when tested with different proteins. Table S7. Primers used in qRT-PCR.
