# Supplementary material for: Putative LysM Effectors Contribute to Fungal Lifestyle
Source: Int J Mol Sci. 2021 Mar 19;22(6):3147. doi: 10.3390/ijms22063147 (PMC8003418; doi:10.3390/ijms22063147)
Supplement: Supplementary file 1 [file ijms-22-03147-s001.zip › Supplementary Material/Supplementary Figure 2.pdf]

PcLys1

A

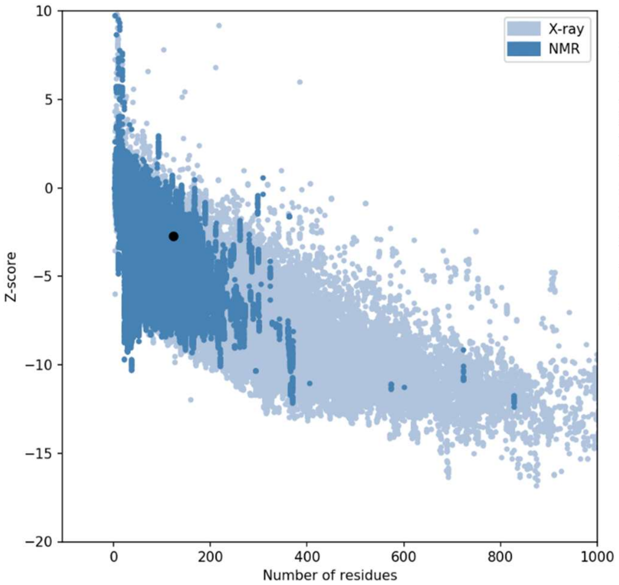

B

```
Residue [   11 :ALA] ( -60.38, 175.72) in Allowed region
Residue [   65 :GLY] (-110.59,  92.99) in Allowed region
Residue [   71 :ASN] (-118.10,  63.84) in Allowed region
Residue [  100 :ASN] ( -98.11,  83.29) in Allowed region
Residue [  104 :GLY] (-176.69,-109.17) in Allowed region
Residue [  110 :LEU] ( -38.59, 130.51) in Allowed region
Residue [   12 :SER] ( -38.00, -23.47) in Outlier region
Residue [   14 :LEU] ( 110.82,  86.34) in Outlier region
Residue [   55 :PRO] ( -22.00,  87.32) in Outlier region
Residue [  115 :TYR] (  69.42, 123.35) in Outlier region
Residue [  119 :GLY] (  45.88, -97.56) in Outlier region
Number of residues in favoured region    (~98.0% expected)   :  110 ( 90.9%)
Number of residues in allowed region     ( ~2.0% expected)   :    6 (  5.0%)
Number of residues in outlier region                         :    5 (  4.1%)
```

PcLys2

C

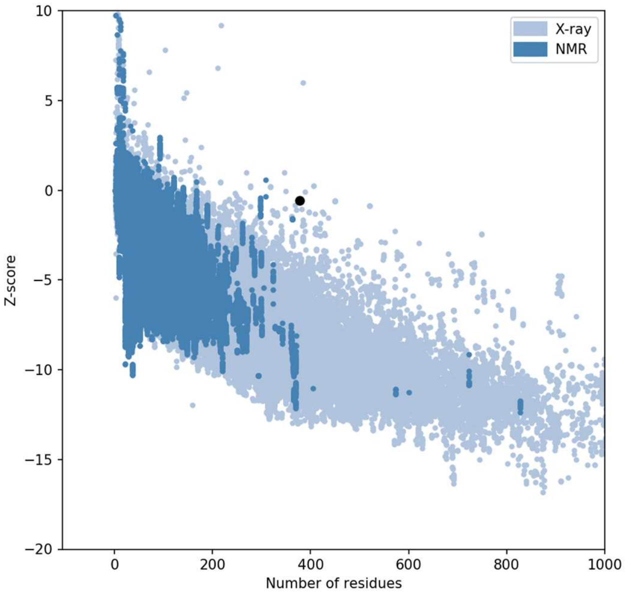

D

```
Residue [   53 :ASP] (-110.30,  44.38) in Allowed region
Residue [   71 :ASP] (-149.91,  -6.62) in Allowed region
Residue [  139 :VAL] ( -57.78, -71.83) in Allowed region
Residue [  140 :GLN] (-164.62, 116.85) in Allowed region
Residue [  147 :LYS] (  67.48, -28.71) in Allowed region
Residue [  177 :GLY] ( 147.05, -28.93) in Allowed region
Residue [  178 :TYR] (-116.33,  71.68) in Allowed region
Residue [  202 :PRO] (-109.87, 169.12) in Allowed region
Residue [  207 :LYS] ( 175.56,-170.53) in Allowed region
Residue [  235 :ASN] (-109.79,  51.38) in Allowed region
Residue [  277 :THR] (-131.76,-159.85) in Allowed region
Residue [  284 :ASP] (  42.58,  67.41) in Allowed region
Residue [  290 :THR] (-144.32,-163.99) in Allowed region
Residue [  337 :LYS] (-137.38,-157.61) in Allowed region
Residue [  339 :TRP] ( -39.41, 118.78) in Allowed region
Residue [  342 :GLY] (-179.33, 109.59) in Allowed region
Residue [  349 :HIS] (-122.73,-143.65) in Allowed region
Residue [  358 :ASP] (  45.16,  68.87) in Allowed region
Residue [   18 :SER] ( 108.89, 168.47) in Outlier region
Residue [  124 :PRO] ( -27.00, 110.28) in Outlier region
Residue [  190 :LYS] (  64.88,  11.41) in Outlier region
Residue [  200 :GLY] (-148.21,  87.16) in Outlier region
Residue [  237 :SER] ( 150.36,-152.55) in Outlier region
Residue [  247 :GLN] (  97.11, 152.65) in Outlier region
Residue [  260 :GLU] ( 163.66, 177.38) in Outlier region
Residue [  269 :CYS] (-161.21,-136.67) in Outlier region
Residue [  270 :THR] ( -59.59,-130.64) in Outlier region
Residue [  357 :ASP] ( 160.57,-115.48) in Outlier region
Residue [  372 :SER] ( 162.19, 168.78) in Outlier region
Number of residues in favoured region    (~98.0% expected)   :  346 ( 92.3%)
Number of residues in allowed region     ( ~2.0% expected)   :   18 (  4.8%)
Number of residues in outlier region                         :   11 (  2.9%)
```

PcLys3

E

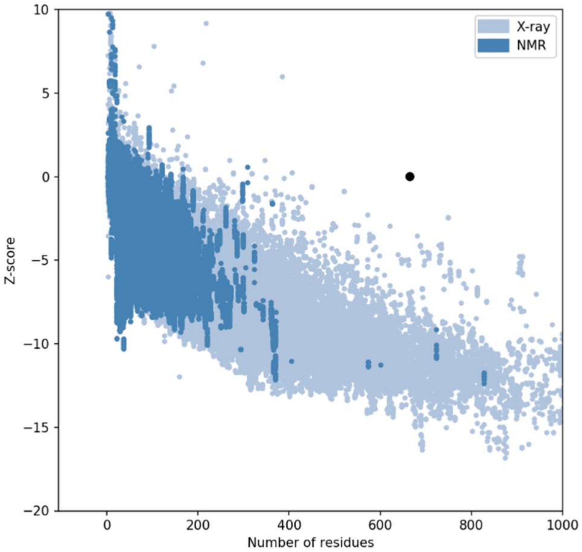

F

```
Residue [   82 :ASP] (-133.73,  71.42) in Allowed region
Residue [   98 :ASP] (-140.58,  40.35) in Allowed region
Residue [  167 :THR] (-104.66, -74.88) in Allowed region
Residue [  168 :TYR] (-176.16, 106.49) in Allowed region
Residue [  176 :CYS] (-134.81,-160.82) in Allowed region
Residue [  213 :GLU] (-136.42,-161.27) in Allowed region
Residue [  215 :CYS] (-117.40,-113.05) in Allowed region
Residue [  235 :THR] (-138.33,-158.82) in Allowed region
Residue [  236 :THR] ( -76.10,-166.84) in Allowed region
Residue [  256 :ASP] ( -85.19,  39.87) in Allowed region
Residue [  259 :GLY] (-122.96,  65.10) in Allowed region
Residue [  263 :CYS] (  74.30,-166.61) in Allowed region
Residue [  276 :LYS] (-167.19,-170.10) in Allowed region
Residue [  296 :GLU] (-135.55,-111.32) in Allowed region
Residue [  323 :THR] (  45.81,  27.40) in Allowed region
Residue [  335 :ASN] (  60.57, -10.16) in Allowed region
Residue [  347 :TYR] (-154.13,-166.35) in Allowed region
Residue [  348 :CYS] ( -59.10, 105.85) in Allowed region
Residue [  356 :GLN] (-110.76,  70.73) in Allowed region
Residue [  360 :GLY] (-162.24, 108.62) in Allowed region
Residue [  379 :THR] ( -81.34,-168.08) in Allowed region
Residue [  380 :SER] ( -57.26, 106.69) in Allowed region
Residue [  393 :PRO] ( -68.11,-173.47) in Allowed region
Residue [  394 :GLY] (-135.76,-110.61) in Allowed region
Residue [  398 :ASN] (  71.35, 146.85) in Allowed region
Residue [  399 :CYS] (-179.92,-164.35) in Allowed region
Residue [  419 :CYS] (-121.60,-123.44) in Allowed region
Residue [  426 :ASN] (-155.98,-138.48) in Allowed region
Residue [  428 :VAL] (-139.21,-141.10) in Allowed region
Residue [  433 :LEU] (-173.80, 116.84) in Allowed region
Residue [  434 :LEU] (-172.11,  96.50) in Allowed region
Residue [  435 :GLN] ( -31.63, 134.82) in Allowed region
Residue [  436 :TRP] (-172.74,-170.89) in Allowed region
Residue [  441 :SER] (-148.07,-151.15) in Allowed region
Residue [  444 :SER] ( -70.84, -69.97) in Allowed region
Residue [  448 :SER] ( -52.57, -70.60) in Allowed region
Residue [  451 :LEU] ( -77.29,  12.30) in Allowed region
Residue [  468 :ASP] (-121.61, -69.09) in Allowed region
Residue [  487 :SER] ( -81.95,-169.34) in Allowed region
Residue [  492 :PHE] (-179.69,-171.85) in Allowed region
Residue [  496 :SER] (  73.28, -22.69) in Allowed region
Residue [  529 :LYS] ( 174.34, 151.79) in Allowed region
Residue [  532 :CYS] ( -64.84, 103.56) in Allowed region
Residue [  533 :THR] (-137.11, -35.29) in Allowed region
Residue [  539 :ASP] (  58.16,   0.63) in Allowed region
Residue [  547 :ARG] ( -49.26, 166.76) in Allowed region
Residue [  564 :LYS] ( 167.41, 169.07) in Allowed region
Residue [  565 :SER] ( -60.13, -65.16) in Allowed region
Residue [  614 :ASN] (-141.64,-152.56) in Allowed region
Residue [  615 :CYS] ( -67.25,  61.98) in Allowed region
Residue [  625 :ASP] ( -66.25, 175.82) in Allowed region
Residue [  663 :VAL] (-155.80,  80.67) in Allowed region
Residue [  164 :CYS] (  59.56, 157.26) in Outlier region
Residue [  165 :GLN] ( 158.75, 176.96) in Outlier region
Residue [  252 :SER] (  99.36, 161.95) in Outlier region
Residue [  288 :PRO] (-117.35,-176.34) in Outlier region
Residue [  294 :ASN] (-173.56, -67.45) in Outlier region
Residue [  303 :LYS] ( 136.01,  99.13) in Outlier region
Residue [  336 :ARG] ( 156.72,-161.33) in Outlier region
Residue [  372 :ARG] (-170.20,-161.71) in Outlier region
Residue [  381 :THR] ( 163.31, 146.42) in Outlier region
Residue [  387 :PRO] ( -55.48,  97.50) in Outlier region
Residue [  391 :TYR] (-167.46,-122.36) in Outlier region
Residue [  395 :THR] ( -33.26,-167.26) in Outlier region
Residue [  400 :SER] ( 166.58,-158.69) in Outlier region
Residue [  430 :VAL] ( 170.81, 131.56) in Outlier region
Residue [  432 :GLN] ( 126.08,-124.92) in Outlier region
Residue [  497 :GLY] (-176.98,  42.56) in Outlier region
Residue [  498 :GLY] (-150.02,  90.40) in Outlier region
Residue [  502 :LEU] (  83.27, 117.84) in Outlier region
Residue [  504 :THR] (-176.03, -78.03) in Outlier region
```

```
Residue [  504 :THR] (-176.03, -78.03) in Outlier region
Residue [  509 :ILE] ( 102.68, 124.64) in Outlier region
Residue [  598 :THR] (  72.24, 133.99) in Outlier region
Residue [  646 :THR] ( -27.91, 137.76) in Outlier region
Residue [  649 :SER] (-163.46,-113.21) in Outlier region
Number of residues in favoured region    (~98.0% expected)   :   588 ( 88.7%)
Number of residues in allowed region     ( ~2.0% expected)   :    52 (  7.8%)
Number of residues in outlier region                         :    23 (  3.5%)
```

PcLys4

G

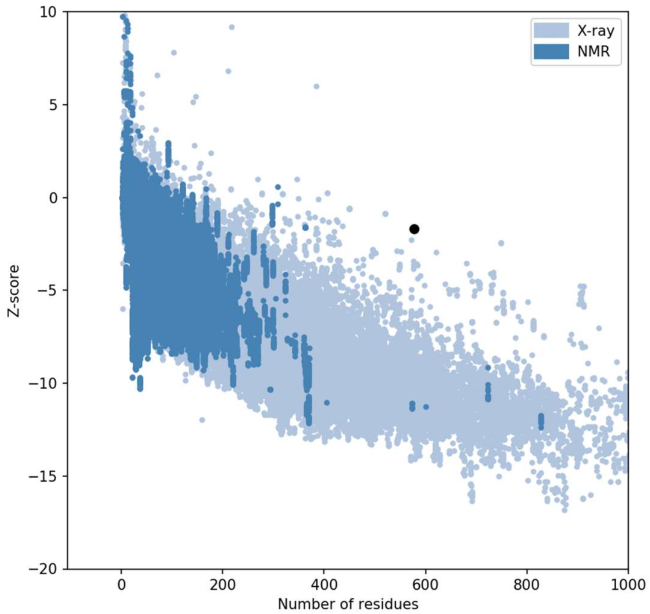

H

```
Residue [  118 :SER] ( -59.71, 168.45) in Allowed region
Residue [  182 :TYR] ( -37.22, 122.87) in Allowed region
Residue [  200 :ALA] (  76.37, -33.40) in Allowed region
Residue [  216 :ASN] (-114.51,  79.89) in Allowed region
Residue [  226 :THR] ( -74.25, -64.50) in Allowed region
Residue [  227 :PRO] ( -96.70, 101.53) in Allowed region
Residue [  229 :VAL] (  45.66,  28.85) in Allowed region
Residue [  249 :CYS] (-145.87,-166.83) in Allowed region
Residue [  256 :GLN] (-165.86,  71.00) in Allowed region
Residue [  276 :PHE] (-173.20,-165.98) in Allowed region
Residue [  277 :PHE] (-154.55, -82.33) in Allowed region
Residue [  290 :LEU] (-145.52,-156.21) in Allowed region
Residue [  296 :TYR] (-132.51,-166.80) in Allowed region
Residue [  318 :GLY] ( -65.55, -76.38) in Allowed region
Residue [  320 :PRO] (-100.87, 143.80) in Allowed region
Residue [  339 :ASP] ( -98.04,  44.45) in Allowed region
Residue [  346 :GLY] (-131.87,  63.26) in Allowed region
Residue [  364 :CYS] (  58.35,-104.34) in Allowed region
Residue [  368 :LYS] (-151.68,-113.64) in Allowed region
Residue [  370 :ASP] (-107.60,  58.11) in Allowed region
Residue [  375 :VAL] (-152.89,-160.10) in Allowed region
Residue [  381 :PRO] ( -71.71, 100.19) in Allowed region
Residue [  388 :LEU] (  69.44, -18.70) in Allowed region
Residue [  407 :LYS] (-143.46,-143.25) in Allowed region
Residue [  415 :SER] ( 174.90, 159.30) in Allowed region
Residue [  416 :ASP] (-125.69,  59.91) in Allowed region
Residue [  450 :ASN] ( -90.73,-165.70) in Allowed region
Residue [  462 :THR] ( -92.93,-151.07) in Allowed region
Residue [  479 :ASN] (-110.92,  62.93) in Allowed region
Residue [  491 :GLY] ( 178.56, 116.37) in Allowed region
Residue [  504 :ILE] (-140.52,-140.74) in Allowed region
Residue [  507 :CYS] (-139.66, -68.83) in Allowed region
Residue [  517 :GLY] ( -38.76, 149.10) in Allowed region
Residue [  519 :SER] (-142.79,-130.95) in Allowed region
Residue [  520 :LEU] ( -53.74, 174.40) in Allowed region
Residue [  557 :CYS] (-105.72,  71.67) in Allowed region
Residue [  558 :VAL] ( -32.97, 104.42) in Allowed region
Residue [  113 :SER] ( -50.10, -88.94) in Outlier region
Residue [  121 :LYS] (  82.64,  -7.04) in Outlier region
Residue [  228 :PRO] ( -38.36,  84.55) in Outlier region
Residue [  264 :ILE] (  39.00,  82.76) in Outlier region
Residue [  274 :GLU] ( 148.38,-127.89) in Outlier region
Residue [  278 :SER] ( 136.25,-173.76) in Outlier region
Residue [  283 :LEU] (  80.11,-158.36) in Outlier region
Residue [  292 :VAL] (  -7.81, 153.58) in Outlier region
Residue [  295 :TYR] (  62.56, 149.01) in Outlier region
Residue [  300 :ASN] ( 174.43,-102.68) in Outlier region
Residue [  302 :ALA] ( 149.86,-153.92) in Outlier region
Residue [  306 :ILE] ( 172.47, -37.99) in Outlier region
Residue [  308 :MET] (-164.42, -24.82) in Outlier region
Residue [  319 :ALA] ( -83.04,-103.49) in Outlier region
Residue [  353 :PHE] (  46.16,-166.87) in Outlier region
Residue [  354 :ILE] ( -32.39, 145.79) in Outlier region
Residue [  357 :ASN] (  82.88, -10.30) in Outlier region
Residue [  362 :GLN] ( -38.38,  72.46) in Outlier region
Residue [  365 :SER] ( -24.28, 133.36) in Outlier region
Residue [  366 :GLY] ( 175.83,  45.32) in Outlier region
Residue [  439 :LEU] (-157.93,-122.31) in Outlier region
Residue [  459 :ALA] ( 169.65,-167.48) in Outlier region
Residue [  463 :ASP] ( -70.90,-141.48) in Outlier region
Residue [  480 :PRO] ( -55.43, -73.48) in Outlier region
Residue [  497 :SER] ( 159.63,-170.06) in Outlier region
Residue [  505 :GLY] ( 164.09,  95.17) in Outlier region
Residue [  509 :ARG] ( 101.87, 110.89) in Outlier region
Residue [  510 :PHE] (-172.33,-129.04) in Outlier region
Residue [  513 :ARG] ( 129.69,-132.73) in Outlier region
Residue [  524 :ASP] ( -47.76,-109.66) in Outlier region
Residue [  549 :SER] (-175.30,-118.53) in Outlier region
Number of residues in favoured region    (~98.0% expected)  :   507 ( 88.2%)
Number of residues in allowed region     ( ~2.0% expected)  :    37 (  6.4%)
Number of residues in outlier region                        :    31 (  5.4%)
```
