# Supplementary material for: Putative LysM Effectors Contribute to Fungal Lifestyle
Source: Int J Mol Sci. 2021 Mar 19;22(6):3147. doi: 10.3390/ijms22063147 (PMC8003418; doi:10.3390/ijms22063147)
Supplement: Supplementary file 1 [file ijms-22-03147-s001.zip › Supplementary Material/Supplementary Tables.pdf]

Supplementary Tables

Supplementary Table 1. NCBI BioProject, Proteome size, Number of proteins containing LysM domains and percentage of proteins containing LysM domains of *P. chlamydosporia* 123 and and 32 other organisms with different lifestyles.

NCBI Bioproject	Organism	Main lifestyle	Proteins with LysM domain(s)
PRJNA571622	*Armillaria mellea*	Phytopathogenic	0
PRJNA41495/PRJNA245119	*Arthrobotrys oligospora* ATCC 24927	Nematophagous	16
PRJNA18467	*Aspergillus clavatus* NRRL 1	Saprophytic	6
PRJNA19263	*Aspergillus niger* CBS 513.88	Saprophytic	12
PRJNA88495	*Aspergillus oryzae* 3.042	Saprophytic	11
PRJNA221345	*Balansia obtecta*	Endophytic	0
PRJNA38719/PRJNA225503	*Beauveria bassiana* ARSEF 2860	Entomopathogenic	17
PRJNA28821	*Blumeria graminis* f. sp. hordei DH14	Phytopathogenic	1
PRJNA183607	*Blumeria graminis* f. sp. tritici 96224	Phytopathogenic	1
PRJNA20061	*Botryotinia fuckeliana* = *Botrytis cinerea* B05.10	Phytopathogenic	5
PRJEA76493	*Claviceps purpurea 20.1*	Phytopathogenic	5
PRJNA37879/PRJNA225514	*Colletotrichum graminicola* M1.001	Phytopathogenic	19
PRJNA47061/PRJNA342684	*Colletotrichum higginsianum* IMI 349063	Phytopathogenic	24
PRJNA41129/PRJNA225510	*Cordyceps militaris* CM01	Entomopathogenic	24
PRJNA277234	*Drechmeria coniospora*	Nematophagous	6
PRJNA593223	*Epichloe coenophiala*	Endophytic	0
PRJNA42133	*Eplchloë festucae* E2368	Endophytic	0
PRJNA243	*Fusarium graminearum* PH-1	Phytopathogenic	14
PRJNA174274	*Fusarium oxysporum* (FOCTR1)	Phytopathogenic	13
PRJNA51499	*Fusarium solani* = *Nectria hematococca* mpVI77-13-4	Phytopathogenic	17
PRJNA360877	*Gymnosporangium juniperus-virginiae*	Phytopathogenic	0
PRJNA352455	*Hirsutella rhossiliensis*	Nematophagous	0

PRJNA242986	*Hypocrella siamensis*	Entomopathogenic	13
PRJNA13835	*Kluyveromyces lactis* NRRL Y-1140	Saprophytic	1
PRJNA29019	*Laccaria bicolor* S238N-H82	Mycorrhizal	8
PRJNA13840/PRJNA1433	*Magnaporthe oryzae* = *Pyricularia oryzae* 70-15	Phytopathogenic	12
PRJNA38715/PRJNA245139	*Metarhizium acridum* CQMa 102	Entomopathogenic	10
PRJNA38717/PRJNA245140	*Metarhizium anisopliae* = *Metarhizium robertsii* ARSEF 23	Entomopathogenic	21
PRJNA132	*Neurospora crassa* OR74A	Saprophytic	9
PRJNA353947	*Peronospora belbahrii*	Phytopathogenic	0
PRJNA391849	*Peronospora destructor*	Phytopathogenic	0
PRJNA453556	*Peronospora effusa*	Phytopathogenic	1
PRJNA285243	*Peronospora tabacina*	Phytopathogenic	0
PRJNA290406	*Phytophthora litchii*	Phytopathogenic	0
PRJEA76339	*Piriformospora indica* = *Serendipita indica*	Endophytic	23
PRJNA327267	*Pleurotus ostreatus*	Nematophagous	8
PRJNA68669	*Pochonia chlamydosporia* 123	Nematophagous	13
PRJNA560630	*Pochonia chlamydosporia* 170	Nematophagous	12
PRJNA398546	*Puccinia coronata* f. sp. avenae	Phytopathogenic	10
PRJNA18535	*Puccinia graminis* f. sp. tritici CRL 75-36-700-3	Phytopathogenic	2
PRJNA661348	*Puccinia kuehnii*	Phytopathogenic	0
PRJNA507656	*Puccinia novopanici*	Phytopathogenic	0
PRJNA277993	*Puccinia sorghi*	Phytopathogenic	2
PRJNA595755	*Puccinia striiformis*	Phytopathogenic	6
PRJNA694214	*Puccinia triticina*	Phytopathogenic	0
PRJNA128	*Saccharomyces cerevisiae* S288C	Saprophytic	0
PRJNA127	*Schizosaccharomyces pombe* 972h-	Saprophytic	1
PRJNA207844	*Serendipita vermifera MAFF 305830*	Endophytic	16
PRJNA19867/PRJNA264112	*Trichoderma atroviride* IMI 206040	Mycopathogenic	16

PRJNA15571/PRJNA225530	*Trichoderma reesei* QM6a	Mycopathogenic	9
PRJNA19983/PRJNA264113	*Trichoderma virens* Gv29-8	Mycopathogenic	20
PRJEA79049	*Ustilago hordei*	Phytopathogenic	2
PRJNA14007	*Ustilago maidis* 521	Phytopathogenic	2
PRJEB25596	*Ustilago trichophora*	Phytopathogenic	3
PRJNA51263	*Verticilium albo-atrum = Verticillium alfalfae* VaMs.102	Phytopathogenic	8
PRJNA28529/PRJNA225532	*Verticillium dhaliae* VdLs.17	Phytopathogenic	10
PRJNA19047	*Zymoseptoria tritici IPO323*	Phytopathogenic	8

Supplementary Table 2. BLASTp of the putative effector Pc123 LysM 1. There are similarities with sequences of entomopathogenic fungi.

Description	Max Score	Total Score	Query cover	E Value	Per. Ident	Accession
hypothetical protein I1G_00006995 [*Pochonia chlamydosporia* 123]	254	254	100%	2.00E-85	100.00	RZR59939.1
hypothetical protein VFPPC_13562 [*Pochonia chlamydosporia* 170]	247	247	100%	2.00E-82	95.93	XP_018144528.1
LysM domain-containing protein [*Metarhizium majus* ARSEF 297]	157	157	98%	3.00E-47	60.98	KID94122.1
LysM domain-containing protein [*Metarhizium robertsii* ARSEF 23]	155	155	98%	2.00E-46	60.16	XP_007824889.1
LysM domain-containing protein [*Metarhizium anisopliae*]	155	155	97%	2.00E-46	60.66	KFG81479.1
hypothetical protein H634G_09367 [*Metarhizium anisopliae* BRIP...	155	155	98%	2.00E-46	60.98	KJK75349.1
LysM domain-containing protein [*Metarhizium guizhouense* ARSEF...	151	151	98%	1.00E-44	58.54	KID84196.1
LysM domain-containing protein [*Metarhizium brunneum* ARSEF 3297]	150	150	99%	3.00E-44	59.68	XP_014540510.1
LysM domain-containing protein [*Metarhizium anisopliae*]	149	149	99%	6.00E-44	59.68	KAF5128564.1
LysM domain-containing protein [*Metarhizium acridum* CQMa 102]	97.4	97.4	50%	1.00E-23	66.13	XP_007812075.1
LysM domain protein [*Metarhizium majus* ARSEF 297]	97.8	97.8	100%	2.00E-23	42.28	KID95850.1
carbohydrate-binding module family 50 protein [*Bipolaris maydi...*	103	307	80%	4.00E-23	52.48	EMD87649.1
LysM domain-containing protein [*Metarhizium robertsii* ARSEF 23]	96.3	96.3	100%	8.00E-23	42.74	XP_007822955.1
LysM domain protein [*Metarhizium guizhouense* ARSEF 977]	96.3	96.3	100%	9.00E-23	42.74	KID83661.1
LysM domain protein [*Metarhizium rileyi* RCEF 4871]	94.4	94.4	100%	3.00E-22	40.65	OAA36463.1
hypothetical protein H634G_05525 [*Metarhizium anisopliae* BRIP...	93.6	93.6	100%	7.00E-22	42.06	KJK79285.1
LysM domain protein [*Metarhizium brunneum* ARSEF 3297]	93.6	93.6	99%	8.00E-22	42.74	XP_014540927.1
LysM domain-containing protein [*Clathrospora elynae*]	93.2	142	80%	1.00E-21	48.00	KAF1939262.1
LysM domain-containing protein [*Metarhizium anisopliae*]	93.2	93.2	99%	1.00E-21	42.74	KAF5137793.1
carbohydrate-binding module family 50 protein [*Alternaria sp....*	95.5	219	80%	1.00E-21	50.50	RII07040.1
LysM domain protein [*Metarhizium anisopliae*]	92.0	92.0	100%	3.00E-21	41.94	KFG79524.1
domain-containing [*Pyrenophora seminiperda* CCB06]	96.7	243	80%	6.00E-21	50.50	RMZ69996.1
hypothetical protein H634G_09266 [*Metarhizium anisopliae* BRIP...	97.4	189	68%	9.00E-21	61.19	KJK75901.1
hypothetical protein CC78DRAFT_470029 [*Didymosphaeria enalia*]	95.1	244	65%	1.00E-20	56.41	KAF2261556.1
LysM domain-containing protein [*Metarhizium anisopliae* ARSEF 549]	96.7	185	69%	2.00E-20	62.69	KID70238.1
LysM domain-containing protein [*Metarhizium majus* ARSEF 297]	95.5	177	58%	2.00E-20	57.75	KID93431.1

hypothetical protein BN1723_017532 [*Verticillium longisporum*]	87.8	87.8	52%	3.00E-20	60.00	CRK17424.1
hypothetical protein AAL_00409 [*Moelleriella libera* RCEF 2490]	95.9	261	60%	3.00E-20	60.29	OAA32944.1
LysM domain-containing protein [*Ophiocordyceps sinensis* CO18]	93.6	93.6	58%	4.00E-20	56.94	EQK98115.1
LysM domain-containing protein [*Metarhizium anisopliae*]	94.0	175	58%	5.00E-20	56.34	KFG84795.1
carbohydrate-binding module family 50 protein [*Bipolaris...*	94.4	301	52%	9.00E-20	64.62	XP_014561981.1
carbohydrate-binding module family 50 protein [*Bipolaris zeico...*	94.4	302	52%	9.00E-20	64.62	XP_007712024.1
hypothetical protein CP532_2862 [*Ophiocordyceps sp....*	87.4	87.4	52%	9.00E-20	56.25	RDA83600.1
hypothetical protein H633G_10937 [*Metarhizium anisopliae BRIP...*	91.7	178	69%	1.00E-19	61.19	KJK85229.1
LysM domain protein [*Metarhizium robertsii ARSEF 23*]	94.0	185	68%	1.00E-19	60.00	XP_007816619.2
hypothetical protein EK21DRAFT_71272 [*Setomelanomma holmii*]	86.7	86.7	52%	2.00E-19	58.46	KAF2027753.1
LysM domain protein [*Metarhizium majus ARSEF 297*]	88.2	88.2	52%	3.00E-19	56.92	KID99357.1
LysM domain-containing protein [*Pochonia chlamydosporia 170*]	92.0	172	52%	3.00E-19	63.08	XP_018138330.1
LysM domain-containing protein [*Colletotrichum siamense*]	91.7	244	65%	3.00E-19	52.38	KAF4806639.1
uncharacterized protein E0L32_002377 [*Phialemoniopsis curvata*]	90.1	145	58%	4.00E-19	58.33	XP_030988592.1
LysM domain protein [*Metarhizium robertsii ARSEF 23*]	91.7	150	52%	4.00E-19	60.00	XP_007816348.1
LysM domain-containing protein [*Colletotrichum siamense*]	91.7	244	65%	4.00E-19	52.38	KAF5497100.1
LysM domain-containing protein [*Metarhizium guizhouense ARSEF...*	87.8	163	69%	4.00E-19	51.19	KID86630.1
LysM domain-containing protein [*Pochonia chlamydosporia 170*]	89.7	154	58%	4.00E-19	55.56	XP_018148099.1
lysM domain-containing protein [*Colletotrichum asianum*]	91.3	242	65%	5.00E-19	51.19	KAF0325696.1
LysM domain-containing protein [*Metarhizium anisopliae*]	91.7	172	58%	5.00E-19	56.34	KFG83697.1
hypothetical protein AG0111_0g3723 [*Alternaria gaisen*]	92.0	274	73%	5.00E-19	60.00	KAB2107892.1
LysM domain-containing protein [*Colletotrichum fructicola*]	91.3	244	58%	5.00E-19	61.54	KAF4907864.1
LysM domain-containing protein [*Colletotrichum fructicola*]	91.3	244	63%	6.00E-19	61.54	XP_031893612.1
hypothetical protein AA0117_g1798 [*Alternaria alternata*]	92.0	274	73%	6.00E-19	60.00	RYN81762.1

Supplementary Table 3. BLASTp of the putative effector Pc123 LysM 2. There are similarities with sequences of entomopathogenic fungi.

Description	Max Score	Total Score	Query cover	E Value	Per. Ident	Accession
hypothetical protein I1G_00011514 [*Pochonia chlamydosporia* 123]	783	783	100%	0.0	100.00	RZR67276.1
hypothetical protein ED733_002102 [*Metarhizium rileyi*]	526	526	100%	0.0	67.19	TWU72501.1
LysM domain-containing protein [*Metarhizium rileyi* RCEF 4871]	525	525	100%	0.0	67.19	OAA42199.1
LysM domain-containing protein [*Metarhizium anisopliae* ARSEF 549]	473	473	90%	3.00E-164	64.58	KID64046.1
hypothetical protein MANI_010103 [*Metarhizium anisopliae*]	433	433	90%	4.00E-149	63.48	KFG84780.1
LysM domain-containing protein [*Metarhizium robertsii* ARSEF 23]	407	612	94%	2.00E-136	56.10	XP_007826696.2
LysM domain protein [*Metarhizium robertsii* ARSEF 23]	393	393	96%	4.00E-132	51.83	XP_007817838.1
LysM domain-containing protein [*Metarhizium album* ARSEF 1941]	347	558	100%	4.00E-115	71.30	KHO00987.1
LysM domain-containing protein [*Beauveria bassiana* ARSEF 2860]	325	325	97%	3.00E-105	44.00	XP_008602669.1
hypothetical protein BB8028_0006g01120 [*Beauveria bassiana*]	323	323	97%	2.00E-104	43.36	PQK15790.1
LysM domain protein [*Metarhizium robertsii*]	323	323	98%	2.00E-104	42.93	EXU97556.1
LysM domain-containing protein [*Beauveria bassiana*]	323	323	97%	2.00E-104	43.75	KAF1738191.1
LysM domain-containing protein [*Cordyceps javanica*]	316	316	97%	5.00E-102	43.80	TQV92266.1
LysM domain-containing protein [*Metarhizium majus* ARSEF 297]	316	316	99%	8.00E-102	41.65	KID93431.1
LysM domain-containing protein [*Cordyceps javanica*]	314	314	95%	6.00E-101	42.01	TQW00740.1
LysM domain-containing protein [*Beauveria bassiana*]	307	307	93%	2.00E-98	43.52	PMB73899.1
hypothetical protein BBAD15_g11618 [*Beauveria bassiana* D1-5]	308	308	93%	9.00E-98	44.01	KGQ03153.1
LysM domain protein [*Metarhizium robertsii* ARSEF 23]	306	306	98%	1.00E-97	37.87	XP_007816291.1
LysM domain-containing protein [*Cordyceps javanica*]	304	304	96%	8.00E-97	40.44	TQV90962.1
hypothetical protein RJ55_03944 [*Drechmeria coniospora*]	300	300	98%	2.00E-95	39.80	ODA80984.1
LysM domain-containing protein [*Drechmeria coniospora*]	300	300	98%	2.00E-95	39.80	KYK61220.1
LysM domain protein [*Metarhizium robertsii* ARSEF 23]	310	310	95%	8.00E-95	42.45	XP_007816619.2
Peptidoglycan-binding lysin domain protein [*Metarhizium rileyi*...	295	295	88%	9.00E-94	43.87	OAA36493.1
LysM domain-containing protein [*Pochonia chlamydosporia* 170]	293	293	87%	9.00E-93	41.55	XP_018138330.1
hypothetical protein G6O67_007750 [*Ophiocordyceps sinensis*]	288	288	83%	4.00E-92	47.47	KAF4505840.1
LysM domain-containing protein [*Metarhizium anisopliae*]	291	291	95%	5.00E-92	37.94	KFG83697.1

LysM domain-containing protein [*Metarhizium brunneum* ARSEF 3297]	286	286	98%	2.00E-90	38.80	XP_014540740.1
LysM domain protein [*Metarhizium robertsii* ARSEF 23]	286	286	84%	3.00E-90	44.25	XP_007816348.1
LysM domain-containing protein [*Metarhizium guizhouense* ARSEF...	285	285	98%	9.00E-90	36.63	KID82718.1
LysM domain-containing protein [*Pochonia chlamydosporia* 170]	270	345	84%	6.00E-86	50.20	XP_018148099.1
LysM domain protein [*Cordyceps fumosorosea* ARSEF 2679]	273	273	68%	1.00E-85	47.60	XP_018700253.1
LysM domain-containing protein [*Pochonia chlamydosporia* 170]	270	270	87%	1.00E-84	41.94	XP_018141296.1
LysM domain-containing protein [*Beauveria bassiana*]	268	353	90%	1.00E-83	48.00	KAF1734749.1
LysM domain-containing protein [*Beauveria bassiana* ARSEF 2860]	268	268	67%	3.00E-83	48.00	XP_008602298.1
LysM domain protein [*Cordyceps militaris*]	266	266	68%	2.00E-82	47.23	ATY61716.1
LysM domain protein [*Cordyceps militaris* CM01]	264	264	68%	8.00E-82	46.86	XP_006674317.1
hypothetical protein H633G_10937 [*Metarhizium anisopliae* BRIP...	258	330	90%	8.00E-81	47.17	KJK85229.1
LysM domain-containing protein [*Ophiocordyceps sinensis* CO18]	256	340	96%	1.00E-79	46.82	EQL03084.1
LysM domain-containing protein [*Metarhizium guizhouense* ARSEF...	256	256	76%	1.00E-79	45.42	KID91348.1
LysM domain protein [*Akanthomyces lecanii* RCEF 1005]	262	422	92%	1.00E-79	39.57	OAA81565.1
hypothetical protein H634G_00888 [*Metarhizium anisopliae* BRIP...	254	330	84%	3.00E-79	47.15	KJK83655.1
LysM domain-containing protein [*Metarhizium anisopliae*]	264	264	79%	3.00E-78	39.88	KAF5126396.1
LysM domain-containing protein [*Metarhizium anisopliae*]	249	249	81%	2.00E-76	40.19	KFG84795.1
LysM domain protein [*Cordyceps militaris*]	245	375	92%	7.00E-73	37.40	ATY67158.1
LysM domain-containing protein [*Cordyceps javanica*]	241	241	89%	1.00E-72	39.39	TQV97459.1
LysM domain protein [*Cordyceps militaris* CM01]	238	238	89%	2.00E-71	36.61	XP_006670025.1
LysM domain protein [*Cordyceps militaris*]	236	236	89%	9.00E-71	36.86	ATY65222.1
LysM domain-containing protein [*Metarhizium anisopliae* ARSEF 549]	240	311	86%	1.00E-69	46.00	KID70238.1
LysM domain protein [*Cordyceps fumosorosea* ARSEF 2679]	224	224	89%	6.00E-66	36.39	XP_018708429.1
hypothetical protein H634G_06422 [*Metarhizium anisopliae* BRIP...	221	221	83%	2.00E-65	35.56	KJK78249.1

Supplementary Table 4. BLASTp of the putative effector Pc123 LysM 3. There are hardly any significant similarities with sequences of other fungi, but saprophytic fungi are among them.

Description	Max Score	Total Score	Query cover	E Value	Per. Ident	Accession
hypothetical protein I1G_00007068 [*Pochonia chlamydosporia* 123]	1363	1363	100%	0.0	100.00	RZR69809.1
hypothetical protein FDECE_9300 [*Fusarium decemcellulare*]	473	521	86%	3.00E-156	48.01	KAF5004162.1
hypothetical protein CNMCM6936_002900 [*Aspergillus lentulus*]	428	428	98%	2.00E-137	38.31	KAF4161886.1
hypothetical protein H101_00782 [*Trichophyton interdigitale* H6]	414	414	99%	3.00E-132	35.46	EZF35683.1
Carbohydrate-binding module family 50 protein [*Trichophyton*...	412	412	97%	3.00E-131	35.81	KAF3898088.1
LysM domain-containing protein [*Beauveria bassiana* ARSEF 2860]	423	423	99%	3.00E-130	36.45	XP_008602577.1
uncharacterized protein ANOM_000100 [*Aspergillus nomiae* NRRL...	399	399	99%	3.00E-125	35.06	XP_015412422.1
LysM domain-containing protein [*Cordyceps fumosorosea* ARSEF 2679]	410	410	99%	3.00E-125	35.70	XP_018705634.1
uncharacterized protein CPUR_06019 [*Claviceps purpurea* 20.1]	391	391	99%	4.00E-123	35.21	CCE32159.1
hypothetical protein DSM5745_03923 [*Aspergillus mulundensis*]	385	385	98%	7.00E-121	35.43	XP_026604935.1
uncharacterized protein CPUR_06013 [*Claviceps purpurea* 20.1]	378	378	98%	4.00E-118	34.87	CCE32153.1
hypothetical protein AFLA70_31g004611 [*Aspergillus flavus* AF70]	374	374	99%	1.00E-115	33.12	KOC07375.1
hypothetical protein COH20_002678 [*Aspergillus flavus*]	371	371	99%	1.00E-114	33.12	RAQ66490.1
LysM domain-containing protein [*Blastomyces dermatitidis* ATCC...	362	411	92%	8.00E-113	35.96	EGE82987.2
LysM domain-containing protein [*Blastomyces gilchristii* SLH14081]	361	410	92%	1.00E-112	35.96	XP_031580869.1
hypothetical protein GE09DRAFT_1090733 [*Coniochaeta sp.* 2T2.1]	360	360	98%	2.00E-110	33.88	KAB5578705.1
hypothetical protein GX50_01673 [*Emmonsia crescens*]	353	400	90%	3.00E-109	35.79	PGH35458.1
hypothetical protein EMCG_05303 [*Emmonsia crescens* UAMH 3008]	352	400	90%	4.00E-109	35.62	KKZ59917.1
uncharacterized protein C285.05 [*Aspergillus udagawae*]	356	472	98%	2.00E-105	35.21	GAO90519.1
hypothetical protein EMPG_12392 [*Blastomyces silverae*]	342	390	90%	3.00E-105	36.79	KLJ12564.1
hypothetical protein CNMCM6936_004141 [*Aspergillus lentulus*]	351	394	98%	8.00E-105	35.12	KAF4160012.1
LysM domain-containing protein [*Colletotrichum graminicola*...	346	346	98%	1.00E-104	33.21	XP_008100462.1
glutamate decarboxylase [*Blastomyces parvus*]	352	400	97%	4.00E-104	34.72	PGH07488.1
LysM domain-containing protein [*Colletotrichum siamense*]	343	343	99%	7.00E-104	32.18	KAF4811551.1
hypothetical protein BDV28DRAFT_132335 [*Aspergillus*...	342	415	94%	2.00E-103	34.93	KAE8353839.1

LysM domain-containing protein [*Beauveria brongniartii* RCEF 3172]	349	349	83%	2.00E-103	37.88	OAA37535.1
uncharacterized protein CGMCC3_g9789 [*Colletotrichum fructicola*]	342	342	99%	2.00E-103	32.40	XP_031883631.1
carbohydrate-binding module family 50 protein [*Lepidopterella...*	340	340	100%	7.00E-103	34.43	OCK74384.1
hypothetical protein GE09DRAFT_1127203 [*Coniochaeta sp.* 2T2.1]	341	525	100%	7.00E-103	33.82	KAB5549646.1
LysM domain-containing protein [*Colletotrichum fructicola*]	341	341	99%	8.00E-103	32.27	KAF4908481.1
carbohydrate-binding module family 50 protein [*Amniculicola...*	337	337	100%	3.00E-102	32.76	KAF2000149.1
carbohydrate-binding module family 50 protein [*Daldinia sp.* EC12]	336	336	98%	1.00E-101	34.18	OTB16721.1
hypothetical protein CA14_011665 [*Aspergillus flavus*]	333	333	83%	2.00E-101	36.69	RMZ37364.1
LysM domain-containing protein [*Colletotrichum gloeosporioides*]	336	336	99%	4.00E-101	32.19	KAF3811932.1
carbohydrate-binding module family 50 protein [*Zopfia rhizophi...*	335	335	98%	5.00E-101	33.20	KAF2175650.1
LysM domain-containing protein [*Colletotrichum siamense*]	335	335	99%	9.00E-101	31.94	KAF5510816.1
carbohydrate-binding module family 50 protein [*Polychaeton cit...*	334	334	97%	2.00E-100	32.73	KAF2716075.1
hypothetical protein PspLS_09235 [*Pyricularia sp.* CBS 133598]	337	550	99%	4.00E-100	40.94	TLD21139.1
hypothetical protein BDZ85DRAFT_301033 [*Elsinoe ampelina*]	333	458	98%	7.00E-100	34.27	KAF2228098.1
hypothetical protein E8E14_003574 [*Neopestalotiopsis sp.* 37M]	329	329	83%	1.00E-99	38.01	KAF3002147.1
hypothetical protein ASPZODRAFT_105672 [*Penicilliopsis zonata...*	327	379	87%	1.00E-99	35.79	XP_022576650.1
carbohydrate-binding module family 50 protein [*Periconia...*	327	486	97%	2.00E-99	36.39	PVI08691.1
hypothetical protein PENSTE_c040G07912 [*Penicillium steckii*]	332	376	93%	2.00E-99	33.44	OQE13960.1
hypothetical protein DL770_005977 [*Monosporascus sp.* CRB-9-2]	330	473	100%	2.00E-98	33.64	RYP81114.1
LysM domain-containing protein [*Colletotrichum siamense*]	327	327	99%	1.00E-97	31.98	KAF4851722.1
hypothetical protein PENANT_c016G05375 [*Penicillium antarcticum*]	327	449	98%	1.00E-97	34.15	OQD83602.1
LysM domain-containing protein [*Colletotrichum aenigma*]	326	326	99%	2.00E-97	32.05	KAF5525599.1
LysM domain-containing protein [*Colletotrichum siamense*]	326	326	99%	3.00E-97	31.90	KAF4875198.1
Peptidoglycan-binding lysin domain [*Penicillium roqueforti* FM164]	326	447	98%	3.00E-97	34.38	CDM37952.1
hypothetical protein PENARI_c008G09124 [*Penicillium arizonense*]	326	370	90%	4.00E-97	35.00	XP_022488860.1

Supplementary Table 5. BLASTp of the putative effector Pc123 LysM 4. There are similarities with sequences of phytopathogenic fungi.

Description	Max Score	Total Score	Query cover	E Value	Per. Ident	Accession
hypothetical protein I1G_00010864 [*Pochonia chlamydosporia* 123]	1161	1161	100%	0.0	100.00	RZR58789.1
lysM domain-containing protein [*Pochonia chlamydosporia* 170]	1124	1124	100%	0.0	95.78	XP_018137526.2
hypothetical protein ASPZODRAFT_162157 [*Penicilliopsis zonata*...	566	630	97%	0.0	52.61	XP_022577051.1
LysM domain-containing protein [*Colletotrichum siamense*]	546	546	95%	0.0	52.02	KAF4837432.1
LysM domain-containing protein [*Colletotrichum gloeosporioides*]	544	741	98%	0.0	51.10	KAF3809926.1
LysM domain-containing protein [*Colletotrichum siamense*]	543	741	99%	0.0	51.61	KAF4823116.1
hypothetical protein CI238_10075 [*Colletotrichum incanum*]	542	623	97%	0.0	52.82	KZL82752.1
LysM domain-containing protein [*Colletotrichum incanum*]	540	666	95%	0.0	52.75	OHW97901.1
LysM domain-containing protein [*Colletotrichum tropicale*]	540	739	99%	0.0	51.43	KAF4835201.1
LysM domain-containing protein [*Colletotrichum siamense*]	540	737	99%	0.0	51.44	KAF5506626.1
LysM domain-containing protein [*Colletotrichum aenigma*]	539	737	97%	0.0	51.44	KAF5524229.1
LysM domain-containing protein [*Colletotrichum gloeosporioides*...	538	680	93%	0.0	51.64	EQB55337.1
carbohydrate-binding protein [*Colletotrichum asianum*]	537	730	99%	0.0	51.35	KAF0327803.1
LysM domain-containing protein [*Colletotrichum viniferum*]	536	727	97%	0.0	49.92	KAF4930678.1
a13e5e8b-a26f-4556-a097-00630ad892c5 [*Thermothielavioides*...	536	729	97%	0.0	52.60	SPQ23050.1
uncharacterized protein CGMCC3_g12747 [*Colletotrichum fructicola*]	535	728	97%	200E-180	49.92	XP_031880753.1
hypothetical protein CHGG_03555 [*Chaetomium globosum* CBS 148.51]	517	517	95%	100E-173	49.22	XP_001230071.1
carbohydrate-binding module family 50 protein...	500	500	95%	300E-167	50.86	XP_003655009.1
hypothetical protein B5807_02521 [*Epicoccum nigrum*]	451	451	99%	100E-148	43.16	OSS52543.1
carbohydrate-binding module family 50 protein [*Bipolaris*...	434	434	99%	800E-142	44.05	XP_014562094.1
hypothetical protein COCC4DRAFT_193590 [*Bipolaris maydis* ATCC...	431	431	99%	100E-140	43.89	XP_014080199.1
LysM domain-containing protein [*Colletotrichum tropicale*]	416	416	97%	200E-135	43.20	KAF4828357.1
carbohydrate-binding module family 50 protein [*Bipolaris*...	416	416	96%	800E-135	39.73	XP_007705696.1
hypothetical protein CI238_06532 [*Colletotrichum incanum*]	412	539	99%	400E-134	41.95	KZL80226.1
carbohydrate-binding module family 50 protein [*Amniculicola*...	413	413	98%	600E-134	39.67	KAF2007436.1
LysM domain-containing protein [*Colletotrichum siamense*]	409	409	99%	100E-132	43.24	KAF4869936.1

hypothetical protein EJ07DRAFT_82962 [*Lizonia empirigonia*]	408	529	96%	100E-132	44.83	KAF1359810.1
lysM domain-containing protein [*Colletotrichum asianum*]	409	409	99%	200E-132	43.57	KAF0325192.1
LysM domain-containing protein [*Colletotrichum incanum*]	408	535	99%	200E-132	41.78	OHW96268.1
LysM domain-containing protein [*Colletotrichum siamense*]	408	408	99%	200E-132	43.57	KAF4844231.1
LysM domain-containing protein [*Colletotrichum aenigma*]	408	408	99%	300E-132	43.41	KAF5526534.1
LysM domain-containing protein [*Colletotrichum siamense*]	405	405	99%	600E-131	43.07	KAF4820443.1
LysM domain-containing protein [*Colletotrichum gloeosporioides*]	399	399	99%	600E-129	42.50	KAF3805423.1
LysM domain-containing protein [*Colletotrichum fructicola* Nara...	399	399	99%	700E-129	42.74	KAF4482207.1
LysM domain-containing protein [*Colletotrichum gloeosporioides*...	399	399	99%	200E-128	42.33	EQB55038.1
LysM domain-containing protein [*Colletotrichum tofieldiae*]	397	665	97%	700E-127	43.46	KZL74226.1
peptidoglycan-binding protein [*Akanthomyces lecanii* RCEF 1005]	390	598	96%	800E-127	47.63	OAA74801.1
hypothetical protein CONLIGDRAFT_719454 [*Coniochaeta ligniaria*...	384	553	99%	800E-122	43.28	OIW22890.1
hypothetical protein E8E14_007637 [*Neopestalotiopsis sp.* 37M]	381	731	99%	900E-121	41.54	KAF3002842.1
LysM domain-containing protein [*Microsporum canis* CBS 113480]	376	482	99%	200E-120	40.42	XP_002848485.1
hypothetical protein V492_08531 [*Pseudogymnoascus sp.* VKM F-4246]	370	622	94%	100E-118	42.89	KFY05463.1
peptidoglycan-binding protein [*Sporothrix insectorum* RCEF 264]	376	459	99%	300E-118	40.70	OAA63388.1
hypothetical protein V494_04919 [*Pseudogymnoascus sp.* VKM F-45...	365	612	94%	200E-116	41.78	KFY36952.1
hypothetical protein CHGG_09900 [*Chaetomium globosum* CBS 148.51]	364	613	99%	600E-115	41.20	XP_001227827.1
carbohydrate-binding module family 50 protein [*Bipolaris zeico*...	360	512	96%	700E-115	45.36	XP_007716749.1
hypothetical protein V490_07063 [*Pseudogymnoascus sp.* VKM F-3557]	356	640	97%	200E-114	49.16	KFX89378.1
hypothetical protein V495_01788 [*Pseudogymnoascus sp.* VKM F-45...	356	640	97%	300E-114	49.16	KFY47843.1
carbohydrate-binding module family 50 protein [*Hypoxylon sp*....	357	478	97%	400E-114	45.88	OTB01250.1
hypothetical protein AJ79_08923 [*Helicocarpus griseus* UAMH5409]	358	476	96%	500E-114	41.88	PGG98286.1
carbohydrate-binding module family 50 protein [*Bipolaris oryza*...	353	461	96%	4,00E-112	44.09	XP_007692779.1

Supplementary Table 6. Percentage of model identity of the 4 putative LysM effectors of Pc123 when tested with different proteins.

Protein-Organism	PDB ID	Pc123 Lys1	Pc123 Lys2	Pc123 Lys3	Pc123 Lys4
Ecp6 *Cladosporium fulvum*	4B8V	21.74%	21.66%	42.00%	55.00%
Chitinase A *Pteris ryukyuensis*	4PXV	39.53%	45.65%	44.66%	38.30%
Chitinase A *Equisetum arvense*	5BUM	32.43%	39.22%	36.73%	27.45%
OsCEBiP *Oryza sativa*	5JCD	-	-	37.04%	22.22%

Supplementary Table 7. Primers used in qRT-PCR.

Name	Primer Sequence	Length
PcLys1b_F	TTGCCAAGACTTTGCTCCTT	149 bp
PcLys1b_R	TCGTTGTCGTTGTTGATGGT	
PcLys2a_F	GGTATCACTCCGGCTTTTGA	72 bp
PcLys2a_R	GCTTCCGCTGGCAATAGTAG	
PcLys3c_F	AACTCCGGCGTTAACAGAGA	235 bp
PcLys3c_R	GTGAAGCTCGTCGAAGGAAC	
PcLys4a_F	ACCCAAAAGACGTCGTCAAC	153 bp
PcLys4a_R	GAGGTGACTGGGTTTCGTGT	
VCP1q_F	GCCATCGTTGAGCAGCAG	
VCP1q_R	ACCGTGACCGTCGTTGTTCT	
Btub_F (HK)	TCCCTCGTCTGCACTTCTTCA	254 bp
Btub_R (HK)	CCATTCGACAAAGTAGGTCGAGTT	
AllPerm_F (HK)	TCGGCATCAACATCATCCTA	94 bp
AllPerm_R (HK)	CCCAGGATGAACCTGACAGT	
GADPH_F (HK)	GCAACACCAACTCCTCCATC	79 bp
GADPH_R (HK)	TACCAGGAGACCAGCTTGAC	

Supplementary Figure Legends

Supplementary Figure 1. LysM domains of putative effectors of different organisms are grouped according to their way of life. Phylogeny of putative LysM effectors belonging to 27 different organisms, only LysM domains (232 in total). Phylogeny is grouped into lifestyles: endophytes, phytopathogens, both and others. Phylogenetic analysis was performed in MEGA X by aligning the sequences using ClustalW, with a Maximum Likelyhood, 1500 Bootstraps, JTT method. Abbreviations are listed in Table 1.

Supplementary Figure 2. Quality of protein models. A, Pc123 Lys1 ProSa; B, Pc123 Lys1 "Rampage" Ramachandran data; C, Pc123 Lys2 ProSa; D, Pc123 Lys2 "Rampage" Ramachandran data; E, Pc123 Lys3 ProSa; B, Pc123 Lys3 "Rampage" Ramachandran data; F, Pc123 Lys4 ProSa; G, Pc123 Lys4 "Rampage" Ramachandran data.

Supplementary Figure 3. Putative Pc123 LysM effectors are expressed in banana. VCP1 (used as positive control) is overexpressed during root colonization. There were not found significant differences in putative LysM expression between banana roots colonized by Pc123 and Pc123 growing in minimal medium.
