# Supplementary figures and images for: Putative LysM Effectors Contribute to Fungal Lifestyle
Source: Int J Mol Sci. 2021 Mar 19;22(6):3147. doi: 10.3390/ijms22063147 (PMC8003418; doi:10.3390/ijms22063147)

- ▼ Phytopathogenic  
■ Both  
▲ Endophytic  
◆ Others

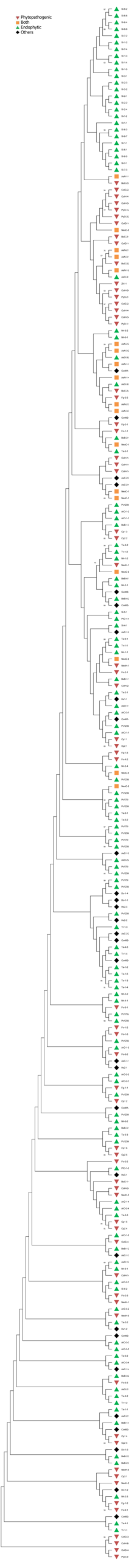

Supplement: Supplementary file 1 [file ijms-22-03147-s001.zip › Supplementary Material/Supplementary Figure 1.pdf]

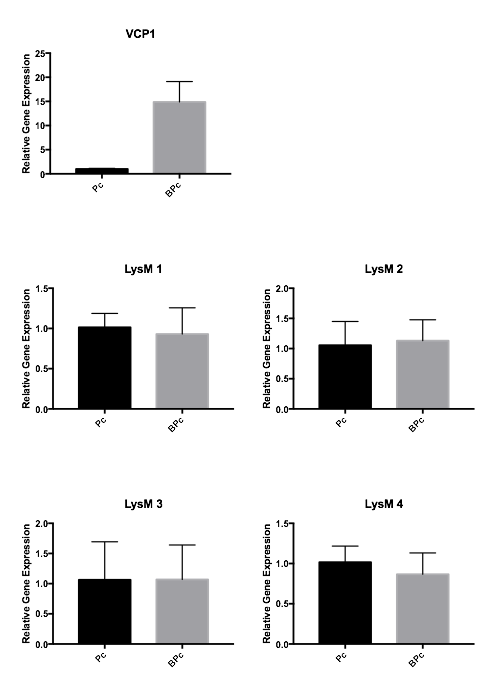

Supplement: Supplementary file 1 [file ijms-22-03147-s001.zip › Supplementary Material/Supplementary Figure 3.png]
